# The Progress of Glucose Monitoring—A Review of Invasive to Minimally and Non-Invasive Techniques, Devices and Sensors

**DOI:** 10.3390/s19040800

**Published:** 2019-02-15

**Authors:** Wilbert Villena Gonzales, Ahmed Toaha Mobashsher, Amin Abbosh

**Affiliations:** School of Information Technology and Electrical Engineering, The University of Queensland, St Lucia, Brisbane 4072, Australia; a.mobashsher@uq.edu.au (A.T.M.); a.abbosh@uq.edu.au (A.A.)

**Keywords:** glucose, non-invasive, minimally-invasive, spectroscopy, continuous monitoring, MARD, FDA, ISO 15197, plasmon resonance, fluorescence, ultrasound, metabolic heat conformation

## Abstract

Current glucose monitoring methods for the ever-increasing number of diabetic people around the world are invasive, painful, time-consuming, and a constant burden for the household budget. The non-invasive glucose monitoring technology overcomes these limitations, for which this topic is significantly being researched and represents an exciting and highly sought after market for many companies. This review aims to offer an up-to-date report on the leading technologies for non-invasive (NI) and minimally-invasive (MI) glucose monitoring sensors, devices currently available in the market, regulatory framework for accuracy assessment, new approaches currently under study by representative groups and developers, and algorithm types for signal enhancement and value prediction. The review also discusses the future trend of glucose detection by analyzing the usage of the different bands in the electromagnetic spectrum. The review concludes that the adoption and use of new technologies for glucose detection is unavoidable and closer to become a reality.

## 1. Introduction

Diabetes is a disease that results from abnormal levels of insulin in the body, due to either a malfunction of the pancreas not producing enough insulin or the cells in the body not using it adequately [1]. Insulin is a hormone that regulates the level of glucose by allowing cells to absorb it from the bloodstream to obtain energy or store it for future use. However, if the level of glucose in the blood remains very low or very high for long periods of time, it could cause hypoglycemia or hyperglycemia, respectively, leading to severe medical conditions, including tissue damage, stroke, kidney failure, blindness and heart disease, among others, and finally, death if left untreated [2]. Deficient production of insulin in the pancreas leads to diabetes type 1, characterized by the sudden drop of glucose levels. On the other hand, ineffective use of insulin leads to diabetes type 2, which is characterized by high levels of glucose. Both conditions do not have a cure, meaning that regular glucose monitoring in diabetic people is necessary for the rest of their lives.

Unfortunately, the issue of regularly checking the blood glucose for most diabetic people is not very pleasant. Conventional devices for glucose monitoring use the electrochemical method [3], which requires a small amount of blood to be drawn out of the body by either finger-pricking or a thin lancelet implanted subcutaneously. The difference between both is that the first only provides a snapshot of the glucose level at one specific point in time and does not require professional assistance, so it is called self-monitoring blood glucose (SMBG) monitoring device. The second provides continuous monitoring, and thus it is called continuous-glucose-monitoring device (CGM). However, both of them not only cause discomfort and pain after repeated use but also pose risks of potential infection and tissue damage [4], resulting in poor patient compliance for daily assigned measurements [5]. Consequently, since the end of last century, there has been a continuous effort for developing non-invasive (NI) devices, i.e., no need of bloodletting, and minimally-invasive (MI), aimed at reducing some of the issues connected with the traditional invasive methods. 

The development of a genuinely non-invasive device for glucose measurement would represent a life-changing factor for millions of patients around the world, allowing them to monitor their glucose level confidently and receiving quick treatment if necessary. It also represents a vast potential market. According to the World Health Organization (WHO), currently there are around 450 million cases of diabetes in the world, and the number could potentially reach 700 million by 2045 [6], with an increase to 39.7 million by 2030 and 60.6 million in 2060 in the United States alone [7]. 

Current developments try to exploit the characteristics of the glucose molecule at different frequencies in the spectrum, from DC and ultrasound, all the way to the near-infrared (NIR) and visible regions. However, it is in these last two where most of the promising technologies have emerged and even been used in the development of some commercial devices. Many are no longer existent due to low accuracy, selectivity and sensitivity of the measurement [8], whereas those already available, still have not reached accuracies comparable to the traditional methods. This situation leaves the issue of NI glucose monitoring still open to many possibilities, including the combination of several techniques, which could finally lead to the development of a reliable and cost-effective device for glucose monitoring.

Many prominent publications have already reviewed several NI glucose technologies and devices, some of which are mentioned hereafter. Chen et al. for example provide a comprehensive description of the current state of MI and NI technologies for CGM analysis [9]. Lin et al. reviewed not only some of the past and current NI devices, but also discuss the main challenges associated with NI detection [10]. Van Enter and von Hauff reviewed the physical and chemical properties of the glucose molecule and analyze their effect on the accuracy and effectiveness of NI technologies [11]. Uwadaira and Ikehata not only provided a comprehensive list of technologies employed for non-invasive glucose detection but also summarize their main advantages and limitations. Khalil provided an excellent account on the properties and characteristics of the glucose molecule and tissue at different NIR wavelengths, and then compares and analyzes the accuracy and sensitivity of glucose measurements in in-vitro, ex-vivo and in-vivo samples [12]. 

**Figure 1:**
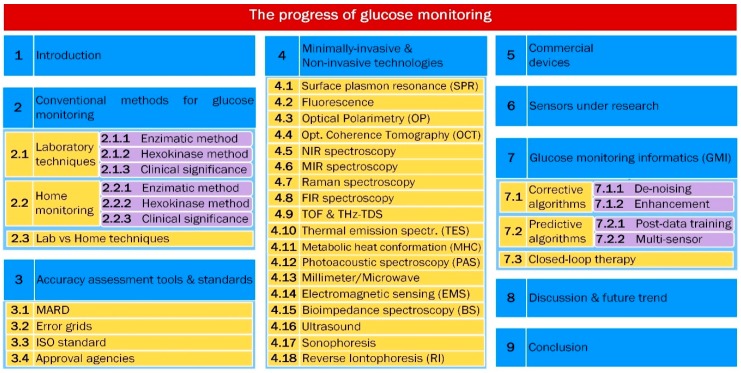
Structure of the paper.

It is upon such previous works that the present review is limited to the topics shown in Figure 1 to provide an update on the technologies behind the current development of sensors for minimal and noninvasive glucose monitoring and to expand our understanding of how current regulatory framework and data processing algorithms shape such development. Section 2 and Section 3 describe the currently accepted methods, standards and regulatory bodies to understand the way they have been shaping the evolution of non-invasive glucose detection. Section 4 provides basic descriptions of past and current leading technologies and associated instrumentation, putting particular emphasis on the most recent and promising technologies, from our perspective. Section 5 lists currently existent devices and those with high potential of coming out to the market shortly (NI and MI), along with the technology and main characteristics associated with each of them. Section 6 lists the techniques currently under research and development in different universities and research institutions. Then, Section 7 provides a brief view of the different types of algorithms used not only to improve the quality of the detected signals but also to predict future values for better treatment of diabetic patients. Section 8 brings together all the technologies, methods, devices and research described in the previous sections, shows them in a graphical chart and discusses the current progress and prospects for further development. Finally, the conclusions presented in Section 9 connect the previous sections and provide a further view into the future development of glucose monitoring.

## 2. Conventional Methods for Glucose Monitoring

Glucose concentration can be determined using either whole blood, plasma, or serum samples, although the last two are preferred because readings from whole blood are usually 15% lower due to the additional water content in the blood cells [13]. As such, standard methods require a certain amount of blood, meaning they are invasive.

Initially, glucose measurement could be performed only in laboratories by taking advantage of the reducing and condensation properties of glucose, but issues associated with non-specificity, toxicity and cross-reaction with other agents quickly phased them out from clinical practice [13]. Hence, present techniques rely on enzymatic and hexokinase methods. Both present high degrees of accuracy, specificity and minimum cross-reaction, but while laboratories use both of them, point-of-care and home monitoring prefer the enzymatic method due to its simplicity and relative affordability. 

### 2.1. Laboratory Techniques

Enzymatic-amperometric and hexokinase are the preferred methods for measuring blood glucose concentrations at laboratories. Table 1 shows some of the equipment based on such methods. 

All of them possess a high level of specificity, sensitivity and can detect a broad range of glucose concentrations, including under hypoglycemic and hyperglycemic scenarios. Such characteristics mean they can be used as the standard of reference to measure the performance of less accurate instruments, i.e., SMBG, CGM and future non-invasive and minimally-invasive devices. Besides, it is important to point out that most of the laboratory equipment can detect and measure other sugars, and chemical compounds, including lactose, methanol and hydrogen peroxide by using other reagents or combined methods, however, this is not discussed in this publication.

#### 2.1.1. Enzymatic-Amperometric Method

**Figure 2:**
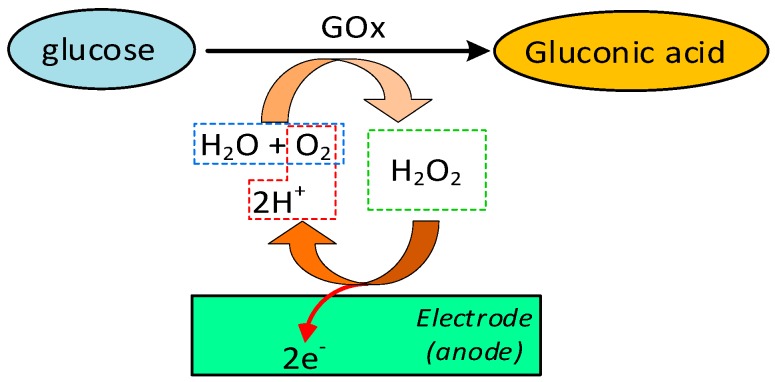
Enzymatic-amperometric method for measurement of glucose concentration in-vitro.

Considering that the enzyme glucose oxidase (GOx) is specific to glucose, in this method, the oxidation of glucose takes place in the presence of GOx, oxygen (O_2_) and water (H_2_O) to form gluconic acid and hydrogen peroxide (H_2_O_2_). The hydrogen peroxide is then electrochemically oxidised at the anode of an electrochemical probe, producing an amperometric signal (current) proportional to the concentration of glucose in the sample (Figure 2). 

A popular glucose analyser using such technology is the blood gas analyser, which contains a solution of GOx between the gas permeable membrane of a pO_2_ electrode and an outer semipermeable membrane. Through diffusion, the glucose crosses the semipermeable membrane and reacts with GOx. Once the hydrogen peroxide is oxidised, the reaction consumes the oxygen near the surface of the pO_2_ electrode, then the consumption rate is measured. The loss of electrons and the rate of decrease in pO_2_ is directly proportional to the concentration of glucose in the sample [20]. 

#### 2.1.2. Hexokinase Method

**Figure 3:**
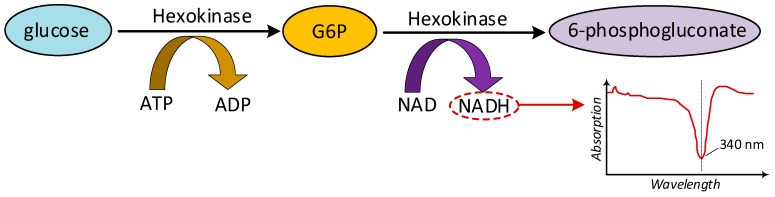
Hexokinase method for measurement of glucose concentration in-vitro.

Hexokinase method, also known as photometric method, consists of a series of chemical reactions, as shown in Figure 3. In the first stage, the glucose reacts with the enzyme hexokinase, in the presence of adenosine triphosphate (ATP) and magnesium ions, to produce glucose-6-phosphate (G6P) and adenosine diphosphate (ADP). In the second stage, G6P and nicotinamide adenine dinucleotide (NAD) go through oxidation with glucose-6-phosphate dehydrogenase until being reduced to 6-phosphogluconate and nicotinamide-adenine-dinucleotide-reduced (NADH), respectively. The amount of NADH is proportional to the amount of glucose in the sample, and it has the property of absorbing light at 340 nm. The amount of absorption is proportional to the amount of NADH, meaning that glucose can be measured using standard spectrophotometric techniques [21].

#### 2.1.3. Clinical Significance

Both methods are highly specific, accurate and sensitive. As such, and depending on the specific technology developed by each manufacturer, some models are used as reference gold-standards for calibration of other glucose meters and at central laboratories. Also, given the small size of some models, it is possible to use them in a point-of-care environment, meaning that medical staff can take quick analyses of ill patients, especially in emergency and intensive care units (ICU). 

The main disadvantages associated with laboratory methods is the inherent invasiveness since all these methods need to be done in-vitro, i.e., blood samples taken from patients; the need of trained laboratory personnel, leading to additional costs; and extended waiting periods of time until receiving laboratory results. Besides, not all laboratory equipment is highly accurate, as revealed by a recent study of Liang et al., showing some blood gas analyzers not complying with the new requirements set by the FDA 2014 draft and 2016 final guidance [22], or not providing accurate readings in hypoglycemia cases, especially in patients with unstable hemodynamics [23].

### 2.2. Home-Monitoring Techniques

There are two types of devices intended for personal use and self-assessment: Non-continuous monitoring (NCGM), and continuous glucose monitoring (CGM). As the name implies, NCGM devices (commonly known as self-monitoring blood glucose SMBG devices) are used to monitor glucose levels only at specific points during the day, with a frequency dependent on diabetes type, diet, medication dosage and clinical condition of the person. On the other hand, CGM devices can monitor glucose levels every few minutes automatically, making possible to monitor rapid changes and trends missed by SMBG testing. Nevertheless, the accuracy and reliability of both systems are suitable in point-of-care and self-assessment situations.

#### 2.2.1. Self-Monitoring Blood Glucose—SMBG

SMBG devices are the typical glucometers requiring finger pricking with a lancet to access the capillary blood. The glucose measurement method is then fundamentally the same electrochemical technique previously described. The main difference, however, is that the complete reaction and detection takes place in a glucose test strip connected to a meter. After putting a drop of the blood sample on the test strip, the glucose oxidizes in the presence of an enzyme to produce a certain amount of current proportional to the glucose level. The electrons then travel to the meter containing a current-to-voltage converter to provide a voltage proportional to the level of glucose. 

**Figure 4:**
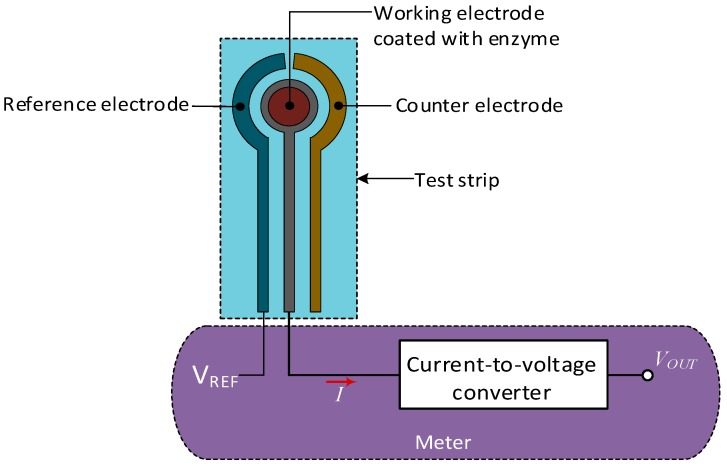
Block diagram of the device for glucose measurement with finger-pricking method.

The test strip contains the enzyme and an arrangement of three electrodes (Figure 4): the working electrode, which senses the actual current of the reaction; the reference electrode, holding a voltage always constant respect to the working electrode to aid with the chemical reaction; and the counter electrode, supplying the current to the working electrode [24]. However, new designs only need the working and reference electrodes. Also, depending on the model, some devices use glucose oxidase GOx as the enzyme, while others use glucose dehydrogenase (GDH) attached to a coenzyme, pyrroloquinoline-quinone PQQ or flavin adenine dinucleotide FDA, although inaccuracy and specificity issues with GDH-PQQ, due to interference with other sugars, is well known, as reported by several studies [25,26,27,28].

#### 2.2.2. Continuous Glucose Monitoring—CGM

CGM devices consist of three essential parts: a wireless receiver, a transmitter and a sensor. The receiver has a monitor displaying the glucose reading. The transmitter is attached to the sensor and transmits the measurements to the receiver via RF waves. The sensor is a tiny sensing device inserted into the subcutaneous tissue, extending just far enough to get access to the interstitial fluid (ISF). Then, by the electrochemical technique, the sensor uses GOx to oxidize the glucose present in the ISF, just as a test strip in SMBG devices does. The resulting peroxide reacts with platinum, to produce the electrical current, which travels along a thin wire to the transmitter, located outside of the skin. Once the receiver gets the data from the transmitter, it processes the information and calculates the glucose level.

#### 2.2.3. Clinical Significance

Even though the apparent advantage of CGM devices is the ability to measure glucose levels continuously, they cannot be considered as the best option for blood glucose monitoring, since they still need calibration at least twice a day with the standard finger-pricking method. Besides, CGM devices measure glucose from the ISF, implying the existence of a lag-time between 6 and 12 min [29], meaning that the ISF readings are not a reflection of the actual glucose level in the blood. Additionally, there are issues associated with inaccurate readings due to mishandling and poor self-monitoring technique, problems related with the insertion of the sensor under the skin, skin irritation and discomfort when securing the device to the body [30,31,32]. 

Finger-pricking SMBG devices are currently the most reliable and accurate devices for self-monitoring due to the relative simplicity of the measuring procedure, and their reliance on capillary blood to obtain accurate glucose readings [31]. Unfortunately, the pain and discomfort caused by regular finger-pricking several times per day, and the costs associated with the constant purchase of test strips, deter many patients from checking their glucose level regularly, as proven by some studies, including one performed by Ward et al in 2015, showing that 50% are willing to measure their glucose levels “occasionally, as needed” [33].

### 2.3. Laboratory Techniques vs Home-Monitoring Techniques

As shown in Table 2, despite the accuracy of both techniques, laboratory and home-monitoring, the invasiveness and time associated are their main limitations. These, not only cause discomfort on patients but also represent potential risks on late diagnostics and sample contamination. However, their level of accuracy and sensitivity still make them the most trusted options for glucose monitoring. 

Laboratory methods are the most accurate and sensitive. Hence they are used as the reference technique to calibrate other devices. Home-monitoring devices are not as accurate as their laboratory counterpart, but still, provide results quickly and accurate enough for personal and point-of-care uses.

## 3. Accuracy Assessment Tools and Standards

In order to test the accuracy and effectiveness of glucose monitoring devices, there is a set of tools, guidelines and standards. The mean-average-relative-measurement (MARD) and the error grids are metric measurements to evaluate accuracy. The standard ISO 15197 provides the quality guidelines, requirements and specifications that glucose measuring devices should comply with to guarantee their suitability for human use. As such, many countries around the world use ISO’s guidelines, through their national agencies, to assess whether each device is suitable for commercialization in their territory or not. Nevertheless, there are exceptions like the United States that has its own set of assessment guidelines. Knowing the metrics and standards is essential to understand the reason why developers and researchers are focusing on certain technologies while leaving behind others, as well as the level of accuracy they intend to achieve. 

### 3.1. Mean Absolute Relative Difference—MARD

MARD is currently the most widely accepted metric measurement to evaluate the performance and accuracy of glucose detection devices, including CGM, SBGM, MI and NI devices, due to its simplicity [34]. Its calculation is quite straightforward, as it consists of taking the average of all the absolute errors between the measured points and those set as the reference. As a result, MARD consists of a single number, expressed as a percentage, which represents the closeness of the measured data to the real value. A small number indicates the capability of the device to take accurate measurements, while large numbers are an indication of considerable inaccuracies. 

The standard way to calculate the MARD is by using two sets of data, taken at the same time, during clinical trials. One set of data is the blood glucose concentration measured by the device under test, while a standard laboratory method provides the second data set (e.g., YSI-2700). Then both measurements are compared [35].

Unfortunately, the value of MARD is heavily dependent on the characteristics and details of the study. Therefore, comparing the MARD of different devices may lead to misinterpretations [36]. As a result, MARD should not be taken blindly as an absolute indicator of accuracy, but rather, as stated by Reiterer et al. [35], as an “indication with some uncertainty”.

### 3.2. Error Grids–Clarke, Parkes and Surveillance

Error grids evaluate the clinical accuracy of glucose measuring devices. They provide a qualitative approach by describing the clinical outcome of basing a treatment decision on the result of the measurement method under evaluation [37]. They consist of a two-dimension grid, divided in a set of ‘risk’ zones’, where results from both, the glucose measuring device and the reference method, are plotted against each other. By analyzing the distribution of paired data points in the grid, it is possible to determine the percentage of points contained in each zone, permitting to categorize each device according to the degree of risk that an adverse measurement would represent due to an inaccurate measurement of the glucose level. 

Clarke error grid (CEG), Parkes or Consensus error grids (PEG) for diabetes types 1 and 2, and Surveillance error grid (SEG) are currently the four main types of error grids, each of them divided into five distribution risk zones identified with letters A to E (CEG and PEG) or a color-coded pattern (SEG). The first one to appear was CEG, but limitations on its assessment method gave way to the development of the PEG which comprises two types of grids, one for each diabetes type, given the higher tolerance of type 2 patients to larger margins of error in the accuracy of the reading than type 1. However, with new regulatory ISO and FDA guidelines, the clinical community becoming more aware of the severe consequences of inaccurate readings [38], and traditional out-of-date medical practices, CEG and PEG are falling out of use, in addition to their inability of identifying clinical states in which tight glycemic control is necessary [39]. As such, in 2014 authors from academia, industry and regulatory agencies introduced the Surveillance Error Grid. Contrary to CEG and PEG, SEG uses different colors from green, for no risk, to red indicating extreme risk of hypo or hyperglycaemia.

Table 3 summarizes the meaning of each zone from a clinical accuracy point of view. In general lines, zones A, B and Green represent accurate or acceptable glucose results, while the ones in the opposite end represent potential dangerous situations if not taking appropriate corrective measures.

### 3.3. ISO 15197 Standard

The International Standards Organization (ISO) is an independent and non-governmental organization that defines and develops specifications for procedures, services and production of high quality, reliable and safe products in a wide range of industries, including medical devices, food safety, environmental management and Information technology among others [42]. Currently, ISO comprises 162 national standard bodies of high technical and expertise levels, and influences regulations of several government agencies worldwide. 

ISO 15197:2013 is the newest standard, released in 2013, for glucose monitoring devices and systems for self-testing. Compared to its ancestor, ISO 15197:2003, the new standard has tighter accuracy requirements that new devices will have to follow. Nevertheless, adhering to the new guidelines will provide greater confidence to patients and clinicians that glucose readings are reliable and sufficiently accurate on a day-to-day basis.

The new standard requires that, compared to a reference laboratory method, 95% of the blood glucose results have to be within ±15 mg/dL for glucose concentrations less than 100 mg/dL or ±15% at glucose concentrations of 100 mg/dL or more. Additionally, 99% of the readings have to be inside of zones A and B of the Parkes (Consensus) Error Grid for diabetes type 1 [43]. 

In 2015, ISO released a harmonized version called EN ISO 15197:2015 to be used by the European Union. This version, however, did not introduce any change to the requirements for the performance evaluation of glucose meters [44,45].

### 3.4. Approval Agencies

Some agencies have their guidelines for the approval of medical devices in their own countries, while others, such as the European Medicines Agency follow the guidelines given by ISO 15197:2013 (devices fulfilling the ISO requirements can get the CE mark) [46]. However, currently there are no specific standards for non-invasive glucose monitors (NIG), as such, manufacturers of NIG devices follow the general guidelines, created for invasive methods, to design their devices and comply with national regulations. 

Table 4 summarizes the evaluation criteria for the acceptance of glucose monitoring devices in certain countries. In the case of countries following the ISO standard, it is important to mention that ISO 15197:2013 is the new standard that new products should comply with if they are released in territories already using the 2013 version. Complying with the requirements from the 2003 version is still accepted in many places. 

## 4. Minimally-Invasive and Non-Invasive Technologies

Technologies for glucose detection without the invasiveness, pain, discomfort and risks associated with standard methods, have been the focus of intensive research. Thus, we can classify them in two major groups: minimally-invasive (MI) and non-invasive (NI). MI technologies are those that need to extract some form of fluid from the body (ex. tears and interstitial fluid) to measure the glucose concentration through an enzymatic reaction. NI technologies rely solely on some form of radiation without the need of accessing to any body fluid.

**Figure 5:**
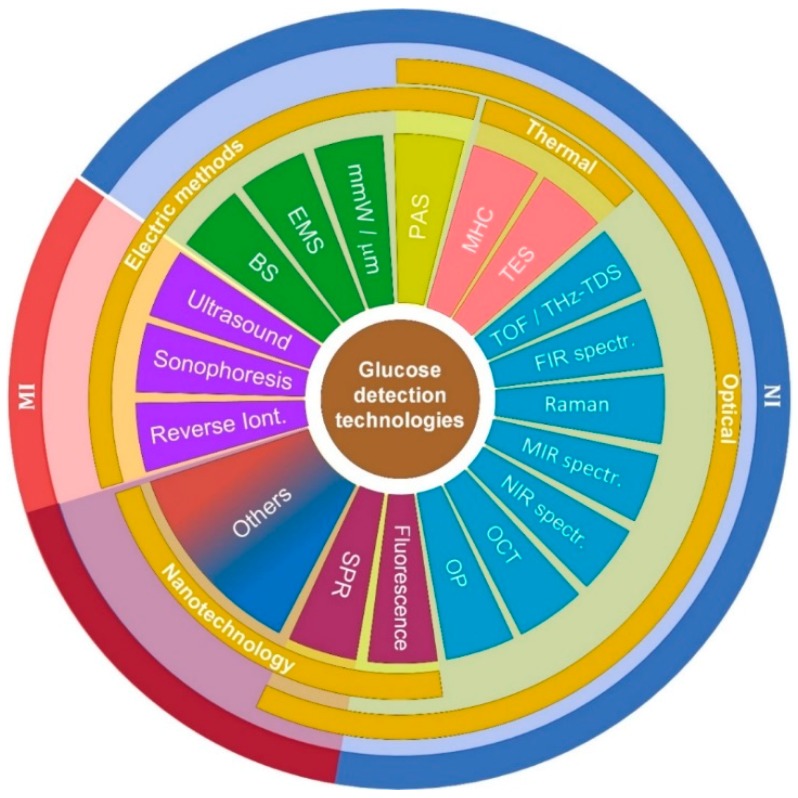
Technologies under development for minimally-invasive and non-invasive glucose detection (SPR-surface plasmon resonance, OP-optical polarimetry, OCT-optical coherence tomography, TOF-time of flight, THz-TDS-Terahertz time domain spectroscopy, TES-thermal spectroscopy, MHC-metabolic heat conformation, PAS-photo-acoustic spectroscopy, mmW-millimeter wave, μm-Microwave, EMS–Electromagnetic sensing, BS-Bioimpedance spectroscopy).

Likewise, technologies for glucose detection can be classified in four sub-groups: Optical, thermal, electrical and nanotechnology methods (see Figure 5). Optical, in a broad sense, comprise all the techniques developed to work in the infrared and optical bands of the spectrum, since they take advantage of the reflection, absorption and scattering properties of light when passing through biological media. Thermal methods monitor glucose by detecting physiologic indices related to metabolic heat generation proper of the glucose molecule, as such, they work in the far-infrared band. Electric methods take advantage of the dielectric properties of glucose at low frequencies using small amounts of electromagnetic radiation, current and ultrasound. Finally, there is the new field of nanotechnology. Currently, only two techniques have started exploring such new venue extensively (SPR and fluorescence), in combination with optical techniques. However, there are several other potential techniques that can be developed, such as carbon nanotubes and plasmonics [58,59,60,61,62], but they are still in a very early stage of development, with most of the progress happening in the theoretical side. As such the authors will not consider them in the present review. Nevertheless, it is important to note that regardless of the type of technology, they all aim at minimizing the influence of physiological variability and various environmental conditions.

### 4.1. Surface Plasmon Resonance (SPR)

Surface plasmon resonance is the point at which the collective coherent charge-density waves, called surface plasmon polaritons (SPPs), are excited by an electromagnetic field radiated onto a thin layer of a highly conductive and chemically inert metal such as gold. The result is an exponentially decaying (evanescent) electric field that is highly sensitive to changes in the refractive index of the surrounding medium (SPR resonance peak). As a result, variations of glucose levels in the sensing medium can be characterized by measuring small changes of refractive index in the interface, and the corresponding shift of the resonant frequency, called ‘SPR shift’ [63], resulting in a shift of the intensity-loss-dip in the SPR reflection intensity curve. 

**Figure 6:**
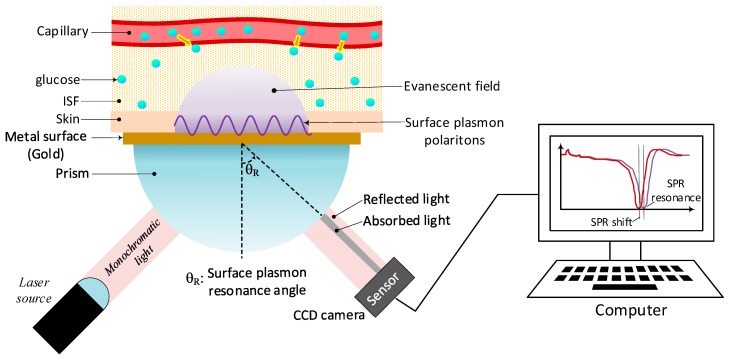
Principle of surface plasmon resonance for glucose monitoring.

As shown in Figure 6, the basic system follows the Kretschmann configuration consisting of a laser source radiating a beam of monochromatic light with transverse-magnetic polarization (TM), also known as p-polarization, through a prism. Under non-resonant conditions, the beam is totally-reflected when it reaches the prism-metal interface, without resonating with the free electrons on the metal layer, leaving behind only an evanescent field made of the electrical component perpendicular to the surface. However, under a particular angle of incidence, known as the resonance angle θ_R_, the momentum of the incoming light equals the momentum of the electromagnetic field produced by the plasma oscillations of the free electrons, causing coupling between the oscillations of the free electrons and the evanescent field. Through such coupling, photons are absorbed through the metal layer, causing a sharp intensity-loss-dip in the reflected power. As a result, the resonance angle characterizes the sample under test given its high dependence on the refractive index of the medium. 

Additionally, SPR also works by radiating the prism-metal interface with polychromatic light at a fixed angle. If the momentum of some particular incoming wavelength matches that of the SPPs, a dip of the light beam at that particular wavelength will appear in the spectrum of the reflected light. The technique is called spectral interrogation mode, and the resonance wavelength is also highly dependent on the refractive index of the surrounding medium [64]. Some other characteristics of SPR technology are shown in Table 5.

SPR technology has significantly evolved during the last years, but mostly focused to the analysis of clinical samples, while the topic of noninvasive detection of glucose has been left behind given the insufficient sensitivity to small concentrations of glucose. As a result, new research is aiming at improving SPR’s specificity by immobilizing different proteins, with good affinity to glucose, on the surface of the metal layer. In this manner, when the glucose solution gets in contact with the surface of the sensor, the protein absorbs the glucose molecules specifically, changing the refractive index of the interface proportionally to the concentration of glucose in the sample [65]. This characteristic makes it suitable as an MI technique if reaching the interstitial fluid becomes feasible. Also, recent advances in the field of nanotechnology have led to the development of a new generation of SPR sensors capable of detecting minimal concentration ranges, in the order of pmol and amol [66]. 

### 4.2. Fluorescence

Fluorescence technology is based on the principle of fluorescent light emission at a specific wavelength after the absorption of radiation of a different energy level, causing a wavelength difference known as Stoke’s shift. The technology makes use of specialized molecules called fluorophores that emit fluorescent light of specific characteristics proportional to the concentration of the analyte under examination.

In the case of glucose, while some of the fluorophores can be bound to the glucose molecule directly, issues associated with low selectivity, irreversibility, interference and analyte depletion, make necessary the use of intermediary molecules called receptors as they bind to glucose more efficiently and can go through reversible changes in their local properties, leading to altered fluorescence [67]. Furthermore, receptors can be of different types and nature including enzymes, boronic acid derivatives, glucose binding proteins (GBPs), and even engineered synthetic materials such as carbon nanotubes [68,69,70] and quantum dots (QDs) [69,71], allowing the use of several fluorescent techniques and monitoring parameters in a broad spectral range, from the ultraviolet (UV) to the near-infrared.

Among the several existing techniques, fluorescence resonant energy transfer (FRET), based on competitive binding-based assays, has received much attention, as it takes advantage of the energy transfer between two light-sensitive molecules called donor (the fluorophore) and acceptor (the receptor). In principle, when glucose binds to the acceptor molecule, the acceptor-donor link is disrupted, leading to decreased electron sharing and increased fluorescence. But, in the absence of glucose, the electron transfer between donor and acceptor increases, leading to less fluorescence [69].

Monitoring of the fluorescent light can be measured either through intensity or decay-time sensing. However, the latter is preferred as the fluorescence lifetime is specific to each analyte, permitting the differentiation between substances, even if they all emit light at precisely the same wavelength [69]. Furthermore, complementing the advantages shown in Table 6, fluorescence lifetime can be precisely measured in scattering media [72], including skin layers [73], indicating that fluorescence technology is suitable for glucose monitoring devices based on transdermal sensing [74], including contact lenses and disconnected transducers inserted into the tissue, commonly known as subcutaneous implants.

### 4.3. Optical Polarimetry (OP)

Optical polarimetry takes advantage of the concept of “chiral molecules”, i.e., molecules that can rotate the plane of polarized light. Glucose is a chiral molecule, as such, it can rotate the polarization plane of a light beam by an angle ‘α‘, in a clockwise direction. The amount of rotation is proportional to the concentration of the analyte, the optical path length, the temperature, and the wavelength of the laser beam, which is usually somewhere between the upper region of the NIR and lower-region of the optical band (~780–400 nm).

**Figure 7:**
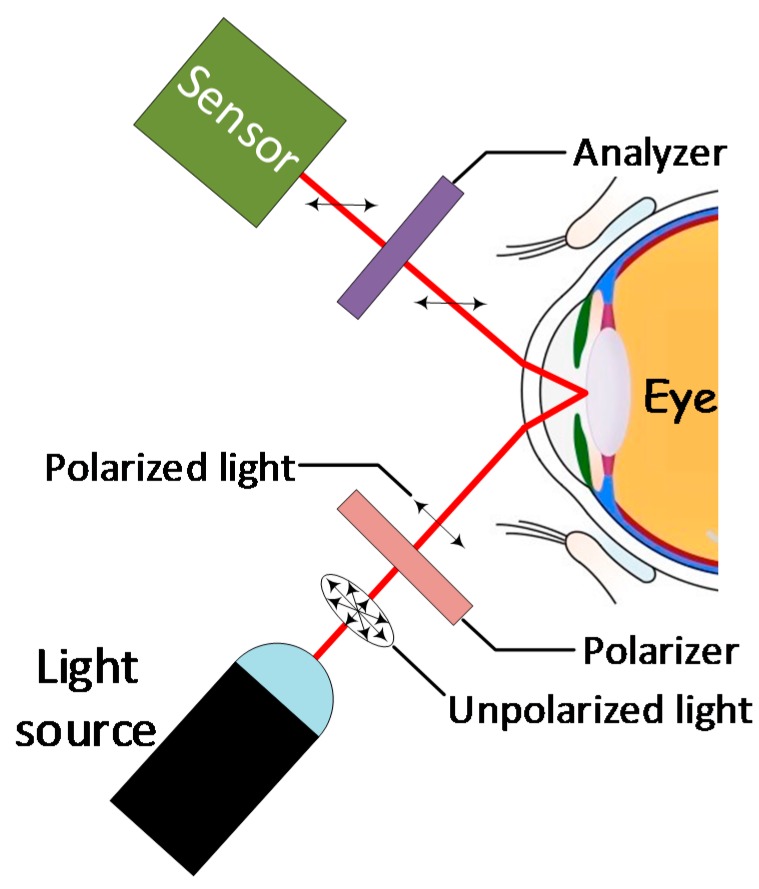
Principle of optical polarimetry in the eye for glucose monitoring.

Unfortunately, the minimal optical rotation associated with physiological level of glucose, the presence of other active molecules, and the high degree of light scattering in the skin and tissue, make it unfeasible to use of optical polarimetry in the skin [75]. However, it is possible to use it on the aqueous humor in the anterior chamber of the eye (Figure 7) due to its excellent optical properties [75,76]. The method consists of polarizing the light emitted by a light source before reaching the eye. The reflected light is then analyzed to determine its angle of rotation α and intensity. Such technique has the potential of detecting small amounts of glucose as long as issues such as sensitivity to temperature and motion and others (Table 7), can be addressed positively.

### 4.4. Optical Coherence Tomography (OCT)

OCT is an imaging technology based on the principles of low coherence interferometry with coherent radiation [77], that is capable of detecting changes of optical characteristics of bio-tissues at micrometer resolutions. Despite being initially developed for tomographic imaging of the eye, it can nowadays measure glucose concentration through the skin with acceptable accuracy and specificity [78]. 

**Figure 8:**
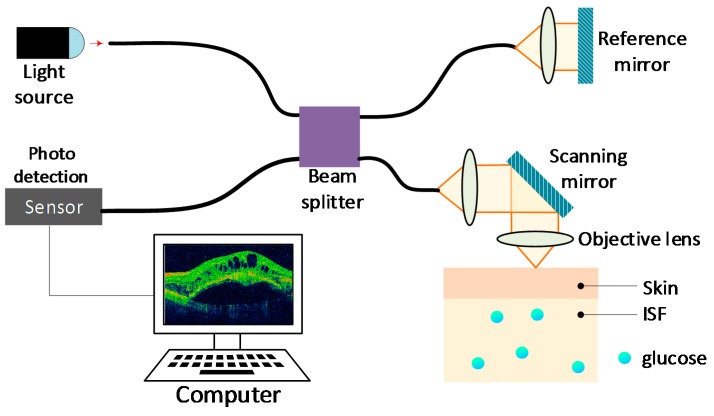
Principle of OCT scanning for glucose monitoring.

The technology consists of radiating the skin with coherent light, with a wavelength between 800 and 1300 nm. The backscattered radiation generated is then combined with a reference to produce an interferometric signal that is sensed by a photodetector, as shown in Figure 8. Hence, if an increase of glucose occurs, it will increase the refractive index and decrease the scattering coefficient, creating a mismatch reduction of the refractive index between the medium and the reference, proportional to the glucose concentration [5]. 

As shown in Table 8, OCT has the great advantage of offering a high signal-to-noise ratio and a high penetration depth, which are very desirable characteristics in non-invasive glucose monitoring. Thus, as long as issues such as temperature change and motion are resolved, this technology possess great promise. 

### 4.5. Near-Infrared Spectroscopy

Near-Infrared spectroscopy (NIRS) technology relies on the absorption and scattering of wavelengths in the 780 nm to 2500 nm range due to molecular vibrations and rotation of bonds inside of the molecule [79]. It uses three basic measurement modes: transmittance, reflectance (including diffuse reflectance), and interactance. However, they all rely on the same core technology, a dispersive spectrometer. 

**Figure 9:**
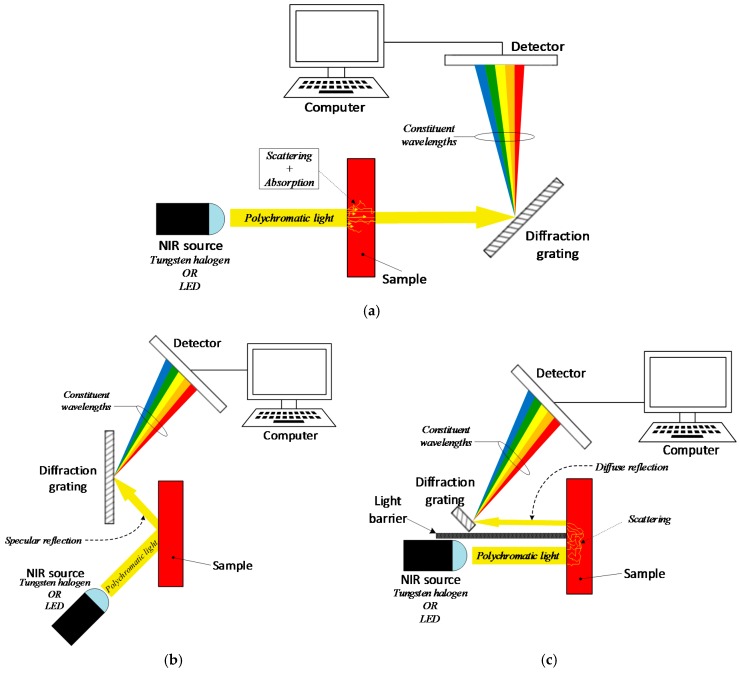
Schematic representation of the three modes of NIR spectroscopy. (**a**) Transmittance mode. (**b**) Reflectance mode. (**c**) Interactance mode.

In transmittance mode (Figure 9a), a light source irradiates polychromatic light onto the sample, and a diffraction grating on the other side splits the transmitted radiation into its constituent wavelengths before being sensed and analyzed by a detector and computer respectively. In reflectance mode (Figure 9b), the diffraction grating and detector are on the same side of the source to detect the specular reflection, i.e., reflection at a definite angle, from the sample. Similarly, interactance mode (Figure 9c), also senses the reflected light from the sample, but it uses a light-barrier between the incident and the reflected beams to separate the field of view of the detector from the illuminated area [80,81]. All modes are suitable for measuring absorption/transmittance and scattering in the sample, and the preference for one of them is based only on the type of media. For example, transmittance mode is preferred for analyzing fluids and very thin or transparent samples, whereas reflectance and interactance, are preferred with dense solids or thick samples.

Although in the NIR band glucose does not present a strong absorption pattern compared to other regions, such as MIR spectroscopy, water also does not absorb much NIR radiation. As a result, up to 95% of light can pass through the stratum corneum and epidermis to reach regions with higher blood concentration, without being affected by skin pigmentation [82]. Also, components and materials for NIR spectroscopy are available in the market at affordable prices. All these advantages, and others listed in Table 5, are leaning developers towards NIR-based technology as the first option to develop self-monitoring non-invasive devices for glucose detection. 

On the downside, as shown in Table 9, NIR presents some disadvantages, including a higher degree of scattering in the tissue and interference of proteins and acids that share similar absorption features with the glucose molecule, leading to increased complexity and unreliability when analyzing the detected signal [83]. As a result, alternative technologies using NIR are getting more attention. One of them is Raman spectroscopy which provides very well defined absorption peaks (see Section 4.7 for further details).

### 4.6. Mid-Infrared Spectroscopy

Mid-infrared spectroscopy (MIRS), also called fingerprint spectroscopy, is a vibrational spectroscopy technique. Hence, it relies on the same system configuration and absorption principles of NIR spectroscopy, but used in the mid-infrared region, approximately between 120 THz (2.5 µm) and 30 THz (10 µm) [84], although some claim 12 THz (25 µm) to be the lower limit in the frequency band [84,85]).

Due to the longer wavelength, there is less scattering of MIR radiation in the tissue, leading to higher absorption rates and specific sharp absorption lines in the spectrum [86], especially between 8–10 µm [87]. This characteristic means that molecules have a unique spectrum in the MIR region, making it ideal for molecular identification. Unfortunately, the strong water absorption in this region does not let MIR signals to penetrate more than some micrometres into the tissue (100 μm approximately) [88], making necessary the use of powerful MIR sources such as Quantum Cascade Lasers (QCL) [89], and the use of complementary technologies, such as photoacoustic spectroscopy (discussed in Section 4.12), to increase the sensitivity towards glucose detection [90]. Table 10 summarizes all these characteristics.

### 4.7. Raman Spectroscopy

Raman scattering determines the degree of scattering of monochromatic light based on the Raman effect. When single-wavelength light hits a target, it produces scattered light travelling in all directions. The majority of this radiation, called elastic or Rayleigh scattering, has the same wavelength as the incident light, while the rest is just a small amount of scattered radiation with a different wavelength, called “inelastic scattering” or “Raman scattering”. Such a wavelength difference is the Raman shift, and it represents the difference between the initial and final vibrational states of the molecule under study [91]. As such, Raman spectroscopy is dependent on the rotational and vibrational states within molecules, and it can be used to detect specific absorption bands and quantify the corresponding molecules [82], meaning that peak locations in the Raman spectrum show the vibrational modes of each functional group within the molecule. Hence, indicating that the Raman shift (expressed in wavenumbers, cm^−1^) will be the same regardless of the wavelength of the incident light. In case of glucose, the most representative vibration modes are those linked to the C—H stretching band, around 2900 cm^−1^, and the C—O and C—C stretching bands between 800 and 1300 cm^−1^ [92,93].

**Figure 10:**
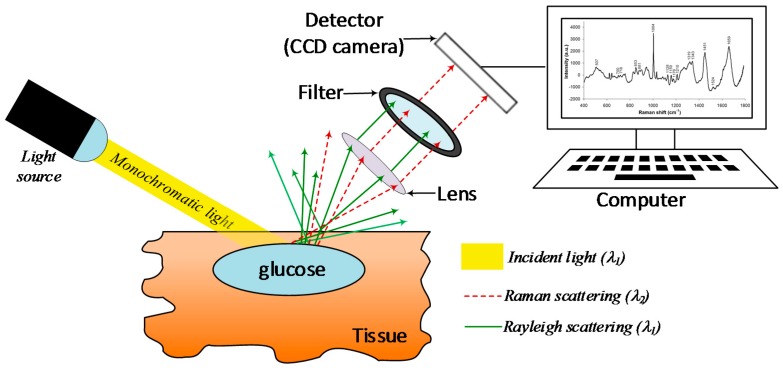
Schematic representation of a basic Raman spectroscopy instrument.

As shown in Figure 10, the basic configuration of a Raman spectrometer consists of a lens capturing part of the scattered radiation and directing it to a filter to let only the Raman scattered light to be sensed by the detector. The computer does the processing of the signal and provides the corresponding Raman shift. Unfortunately, interference and instability issues, as shown in Table 11, prevent Raman spectroscopy from providing accurate glucose measurements in-vivo.

Due to poor depth penetration in the MIR band, most of the study of glucose detection with Raman spectroscopy takes place in the low-frequency end of the NIR band despite the presence of broader spectral features causing interference and variability in the spectrum of the detected signal. For example, the motion of blood corpuscles and other analytes within the analyzed region, tissue autofluorescence, and photobleaching generate strong interference spectra hindering the identification of the glucose signal. Similarly, as stated by Pandey et al., variations due to turbidity in the analyzed volume introduce nonglucose specific variance in the detected spectra, making calibration extremely challenging [94]. As a result, current efforts on minimization or compensation of such issues cover a wide range of techniques and technologies, from multivariate calibration (MVC) analysis to tissue modulation [95] and photon migration theory [94].

### 4.8. Far-Infrared Spectroscopy

FIR spectroscopy, commonly known as Terahertz (THz) spectroscopy, is based on the principle of absorption due to the existence of particular vibrational and rotational transitions of weak bonds and bonds of heavy atoms [96], approximately between 0.3 THz (1000 μm) and 30 THz (10 μm). As seen from Table 12, the lack of information of FIR means that the technology is still in its infancy concerning the field of non-invasive glucose detection since the strong absorption of water and the low levels of power delivered by terahertz sources do not allow the detection of meaningful data using standard NIR and MIR methods. Nevertheless, with the advent of quantum-cascade lasers (QCLs), now it is possible to use time-resolved far-infrared spectroscopy (also called Terahertz time-domain spectroscopy THz-TDS) for biomedical applications in the sub-millimeter wavelength regime (see Section 4.9 for further details).

### 4.9. Time of Flight (TOF) and Terahertz Time-Domain Spectroscopy (THz-TDS)

TOF uses single-frequency very short laser pulses (in the order of picoseconds) to measure the radiation absorption, and time it takes photons to travel across the sample. It uses the same spectroscopic principles of absorption and scattering, but from a time-domain perspective to get the phase change as an additional parameter. When light propagates through the sample, some photons will follow a direct path towards the detector, others will follow a longer zigzagging path due to multiple internal reflections, and others will go through total scattering giving rise to diffuse light. Analyzing the time of flight distribution of detected photons, the changes in the pulse shape (pulse broadening due to scattering), and the absorption level, it is possible to detect the optical properties of the medium, including glucose concentration.

Alraouso et al. describe a typical setup for measuring glucose in-vitro with TOF using 35 picosecond pulses, with a wavelength of 905 nm, hitting a sample, and an array of five optical fibers collecting the scattered light from the medium and directing them towards a camera to record the temporal profiles of the detected pulses [97]. Besides Alraouso’s measurements, TOF needs further study on glucose monitoring.

THz-TDS is similar to TOF as it also uses ultrashort pulses in the time domain (a few hundreds of femtoseconds) to measure the travel time (phase information) of the reflected and scattered signals, and absorption of the medium. However, THz-TDS is unique in its way of measurement as it generally uses an ultrafast-laser pump with a specific pulse-shape (Gaussian or differentiated Gaussian for example) allowing a broad frequency sweep. Hence, permitting spectroscopic information within the detected signal, as well as the possibility of measuring the refractive index and the spectrum of the complex permittivity in a wide frequency range with a single scan. Furthermore, using special processing techniques in the time-domain, it is possible to extract crucial frequency-dependent information such as dynamic range, bandwidth and signal-to-noise ratio [98].

As in many other technologies, the two main modes of operation of THz-TDS are reflection and transmission. However, due to the high level of water absorption, transmission mode has not been able to provide satisfactory results in the THz band. Instead, reflection mode has been the focus of research in the last years, especially between 0.1 and 1 THz in order to take advantage of particular vibrational-rotational transitions of active macromolecules in the blood [99]. Nevertheless, the low depth penetration remains still as a significant obstacle since THz radiation can hardly reach regions underneath the skin containing significant amounts of blood for analysis. Hence, current efforts focus on analyzing the skin in order to correlate changes in its optical characteristics with changes in the glucose level, as existing evidence suggests that changes in glucose levels cause internal physiological changes affecting the skin, including blood osmolarity level, fluid loss in the cells and aggregation of erythrocytes, especially in cases of hyperglycaemia [100]. Such evidence indicates the feasibility of overcoming the water absorption issues associated with standard spectroscopic techniques in the 0.1 to 1 THz band [101,102]. 

As shown in Table 13, the common advantage between TOF and THz-TDS is their immunity to background noise. However, there are still issues related to the long measurement time and the low spatial resolution that require additional investigation.

### 4.10. Thermal Emission Spectroscopy (TES)

**Figure 11:**
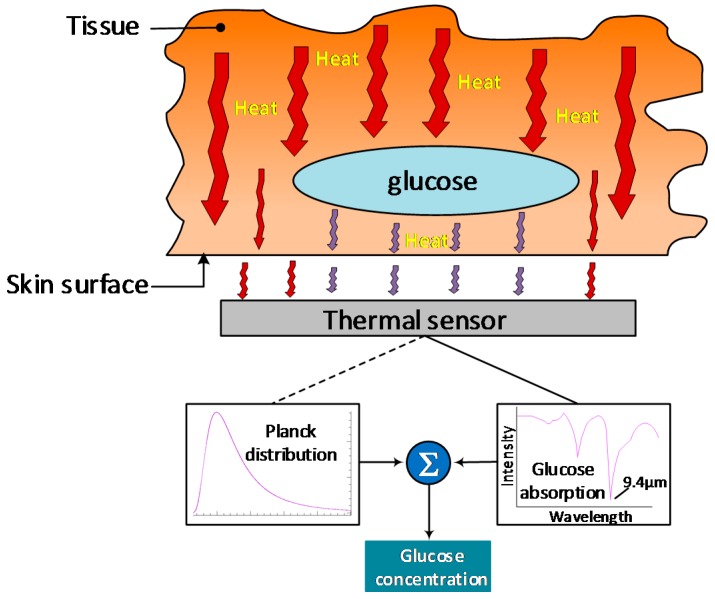
Thermal emission spectroscopy principle.

TES uses the principle that the human body naturally emits energy as heat in the far-infrared band, between 8 μm and 14 μm [103]. During the process of leaving the body, part of this radiation is absorbed by different molecules in the body, including glucose around the 9.4 μm wavelength, meaning that the analysis of the intensity and characteristics of such radiation (Figure 11), provides useful information on the presence and concentration levels of glucose in the tissue [104] with reasonable specificity.

However, just reading and interpreting the radiation absorbed by glucose is not enough to provide accurate readings due to the small amounts of thermal energy involved. Thus, TES also compares the reading with a predicted amount of thermal energy using the Planck distribution function, in which the measured data is the intensity reference level upon which the actual thermal absorption is calculated and then, converted to glucose concentration level [104]. Unfortunately, although in theory, this technology seems to be straightforward, no significant research has been put into it, as such there are several limitations (Table 14) that need to be addressed. The most acknowledged experiment using TES is the one performed by Buchert, back in 2004 [105], in the tympanic membrane, but actual in-vivo results need yet to be published. 

### 4.11. Metabolic Heat Conformation (MHC)

MHC technology consists of measuring the glucose concentration level by measuring physiological parameters associated with the generation of metabolic heat and local oxygen supply [106]. The technique relies on the fact that the metabolic oxidation of glucose not only produces most of the necessary energy for all cellular activities but also generates a certain amount of heat as a byproduct that correlates with the amount of glucose and oxygen levels in the body. The heat emitted to the environment can be in the form of radiation, convection and evaporation. The heat emitted as radiation and convection is linked to the skin and ambient temperatures, whereas the heat dissipated by evaporation, represents the amount of evaporation from the skin [107]. 

**Figure 12:**
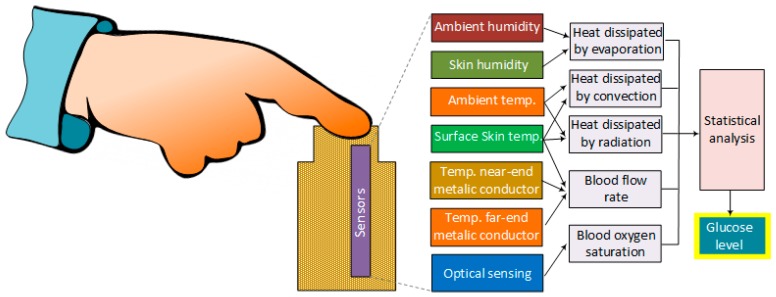
Glucose measurement using MHC (concept taken from [107]).

The parameters recorded by the sensors include thermal generation, hemoglobin (Hb), oxyhemoglobin concentration (O_2_Hb), and blood flow rate. They are all measured in the fingertip by multi-wavelength spectroscopy methods, along with temperatures in the fingertip, ambient and background radiation. The data is then analyzed with different statistical tools, including regression, multivariate and discriminant analyses (Figure 12). However, as shown in Table 15, this technique is also sensitive to interference from temperature variations and sweat.

### 4.12. Photoacoustic Spectroscopy (PAS)

This technology uses the same idea of ultrasound waves, but it employs short laser pulses with a wavelength that is absorbed by a specific molecule in the fluid to produce microscopic localized heating, dependent on the specific heat capacity of the tissue under examination [82]. The absorbed heat causes a volumetric expansion of the medium, generating an ultrasound wave that can be detected by an acoustic or pressure sensor. By tracking the peak-to-peak variations of the detected signal, it is possible to correlate them with the variations of glucose level in the blood.

For noninvasive detection of glucose, pulsed and continuous-wave (CW) are the two main forms of excitation. In pulsed-mode, the pulses have durations in the range of nanoseconds, and a pulse-repetition rate of a few kilohertz, leading to a fast and adiabatic thermal expansion of the sample and generating a wide spectrum of acoustic frequencies [109], along with jitter and acoustic noise in the wide bandwidth of the detector (transducer) [110]. On the other hand, CW excitation employs a modulated continuous wave, generating a single acoustic frequency in the detected spectrum, as well as a higher signal-to-noise ratio if used in a lock-in detection configuration [110].

**Figure 13:**
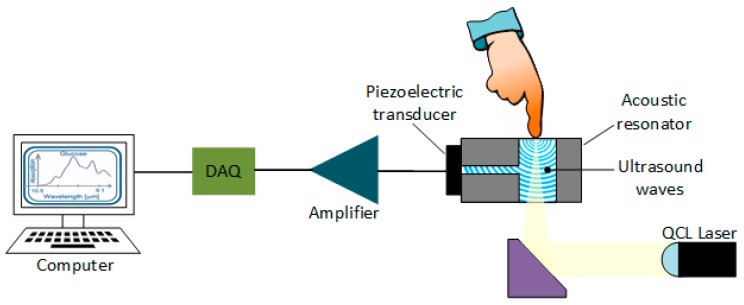
Basic optical setup for noninvasive photoacoustic measurement of glucose.

Figure 13 shows the basic configuration of PAS sensing. The light emitted by a laser impacts on the sample to generate the ultrasound wave, by the process previously discussed. The generated ultrasonic wave propagates through the acoustic resonator, also known as cell, and reaches the detector, which generally consists of a piezoelectric transducer such as a microphone. The electrical signal at the output of the sensor is subsequently amplified, digitized and sent to the computer for analysis. This configuration, however, has the main drawback of poor sensitivity for in-vivo detection of glucose. So, as suggested by Kottmann et al. [111], an alternative to this method is using two laser sources. One covering wavelengths of strong glucose absorption, and the other covering regions insensitive to glucose, in order to obtain a large ratio between the two measurements, improving the overall SNR of the system. Currently the configuration has provided good stability, but the sensitivity is still low, although it can be improved by increasing the power of the laser [111]. 

In addition to the advantages shown in Table 16, PAS can use a wide range of wavelengths, from ultraviolet to NIR. However, it has not been until recently that tests have shown that PAS can even be used in the MIR band. This development allows us to take advantage of the strong absorption characteristics of the glucose molecule between 800–1200 cm^−1^, despite its low penetration depth into human skin (8.33–12.5 μm), thanks to particular vibration modes of the stretching and bending C—H—O band [112]. As a result, current efforts are focusing on the use of QCLs to improve the SNR in parts of the body in which it is feasible to reach the interstitial fluid, i.e., 10–50 μm [113].

### 4.13. Millimeter and Microwave Sensing

Microwave and millimeter radiation present lower energy per photon and less scattering in the tissue [114], indicating that they can go deeper into the tissue to reach regions with sufficient blood concentration, yielding more accurate glucose readings. To take advantage of such characteristic, millimeter and microwave techniques widely used in several areas; including communications, detection and medicine; exploit the reflection, transmission and absorption characteristics of tissues and blood in those bands to correlate their permittivity and conductivity with the concentration of glucose in the body. As a result, there are four basic techniques: Reflection, transmission, resonant perturbation [115], and radar [116].

Reflection methods are one-port techniques, and they aim at measuring the reflection parameter S_11_ to identify the amplitude and phase variation in the reflected signal due to the change of permittivity in the blood when the glucose level varies. The measurement takes place over a wide frequency band utilizing a vector network analyzer connected to a sensor, which, depending on the frequency band under examination, it can be a wideband antenna, an open-ended coaxial line or waveguide. Nevertheless, the principle of operation is the same. The near-field of the antenna or the fringing field of the open-ended line penetrate the upper skin layers to reach depths in which the amount of blood is high enough to sense changes of its permittivity, which are interpreted as changes of the impedance or admittance seen by the sensor, thus varying proportionally S_11_.

Transmission methods are an extension of reflection methods since they provide the full set of S-parameters, allowing the calculation of the complex propagation constant of the media and hence, the attenuation and phase insertion of the transmitted signal due to the variation of glucose levels. However, its accuracy is limited in low loss environments [115]. The transmission method is in essence a two-port technique. Thus, it employs hardware similar to reflection methods in a duplicated configuration. 

The resonant perturbation method is a subset of the reflection and transmission methods in the sense that it uses a near-field sensor with a very high quality-factor Q. The aim is to measure changes of the resonant frequency, the quality factor and the 3dB bandwidth and correlate them with variations of the dielectric properties of the media under test [115]. As such, sensors in the resonant perturbation method operate in a very narrow frequency range. Examples of such sensors are microstrip patch antennas and waveguides [117,118,119], split-ring resonators [115,120], high-Q resonance [121], and dielectric resonator antennas (DRA) [122]. 

Radar technique, contrary to reflection, transmission and resonant perturbation methods, it operates in the far field by sending the electromagnetic wave to the target, located at a certain distance from the transmitter, and waiting for the reflected signal to reach the receiver. The received signal contains not only information about the speed, location, and the extent of the object but also, its composition. Thus, by signal processing and AI algorithms, researches are expecting to find a correlation with the concentration of glucose in the blood [116]. 

As shown in Table 17, all mm-Wave and microwave sensing techniques present some disadvantages. However, the possibility of going deeper into the tissue, avoiding issues proper of optical techniques, is motivating several groups to continue further development in the area.

### 4.14. Electromagnetic Sensing

**Figure 14:**
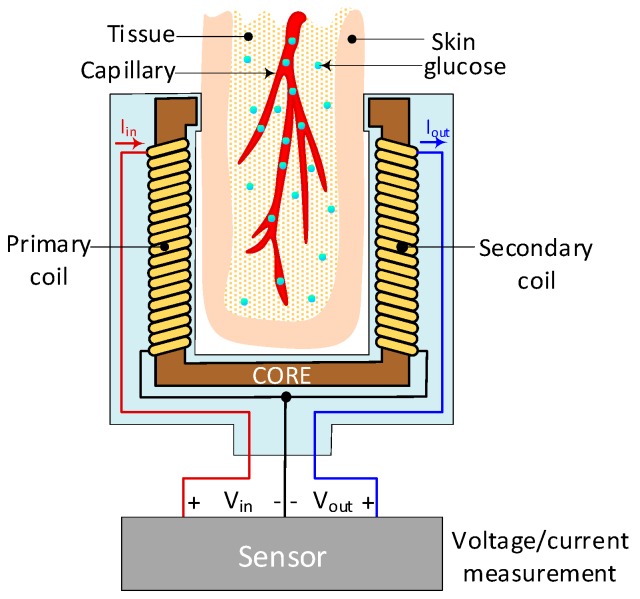
Concept for measuring glucose concentration in the ear lobe using electromagnetic sensing.

This technology measures the current, or voltage, which is proportional to the magnetic coupling between two inductors [8,124]. Since the coupling depends on the dielectric characteristics of the media between the two coils, it is also proportional to the concentration and type of analyte (Figure 14). In other words, the ratio between input and output voltages, or between currents, is proportional to the concentration of glucose. Furthermore, the frequency of the signal plays a fundamental role to produce enough coupling, although this is also dependent on the temperature of the sample under examination. As a result, frequencies in the band between 2.4 MHz and 2.9 MHz are generally accepted to be suitable for the detection of glucose variations in-vivo [4], while others, such as Melikyan et al, suggest that 7.7 GHz is a better option [125]. Additional characteristics are shown in Table 18.

### 4.15. Bioimpedance Spectroscopy (BS)

Also known as dielectric impedance spectroscopy, this technology assesses the changes induced by blood glucose variations in the permittivity and conductivity of the membrane in red blood cells (RBCs). BS uses the concept that variations in plasma glucose concentration induces variations in the concentrations of sodium (Na^+^) and potassium (K^+^) ions, causing changes in the conductivity of the RBCs’ membrane [126], indicating a direct relationship between both. As such, BS applies a small amount of alternating current, of known intensity, to measure the associated resistance and thus, the conductivity. This means the technique is relatively simple, making it potentially affordable and easy to employ in a practical scenario as long as sensitivity to temperature variations and sweat, among other limitations (Table 19) can be minimized. 

### 4.16. Ultrasound

This technology measures the propagation time of ultrasound waves through the media. The higher the glucose concentration, the faster the ultrasonic wave propagates through the media, reducing the time of propagation. Depending on the strength of intermolecular bonding forces and the density of the medium, the fluid or tissue has a certain degree of compressibility which determines the acoustic velocity of low-frequency waves through the media [127]. As such, changes of the glucose concentration in the extracellular fluid affect density and adiabatic compressibility, affecting the acoustic impedance linearly. Other advantages and limitations are shown in Table 20.

### 4.17. Sonophoresis

This technology relies on obtaining a sample of the interstitial fluid to measure the glucose by enzymatic method. The difference is that sonophoresis uses low-frequency pressure waves to drive glucose molecules out through the skin. It relies on the longitudinal nature of ultrasound waves, i.e., the direction of propagation being the same as the direction of oscillation [128], to enhance the permeability of the skin and induce a phenomenon called ‘cavitation’. The working principle of cavitation is not completely well-understood, but it consists of a series of compression and expansion movements of sufficient magnitude to extract gas out from the tissue, carrying with it other permeants, including glucose [129]. However, although this technology is theoretically feasible (as shown by several advantages in Table 21), at present most of the studies are focusing on drug delivery rather than glucose measurement.

### 4.18. Reverse Iontophoresis (RI)

**Figure 15:**
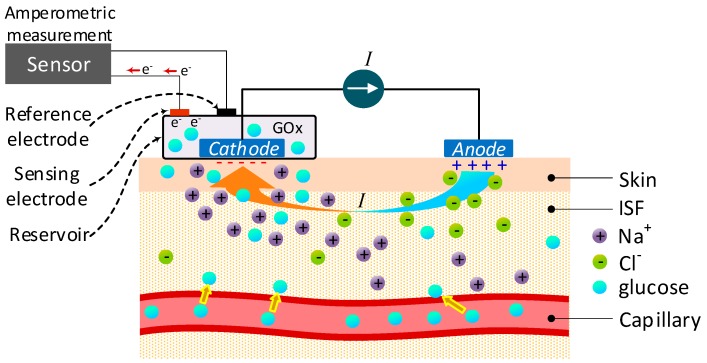
Principle of reverse iontophoresis for glucose monitoring.

Reverse iontophoresis is categorized as a “minimally invasive” technology since it relies on the circulation of a small electric current between an anode and cathode located on the surface of the skin to get access to a small amount of interstitial fluid (ISF). The migration of sodium ions primarily produces the current, causing a convective flow (electro-osmotic flow) of the interstitial fluid (ISF), carrying with it glucose molecules towards the cathode [4]. At the cathode, there is a standard glucose sensor measuring the glucose concentration directly by the enzymatic method, i.e., oxidization by an enzyme, such as glucose oxidase (GOx) as shown in Figure 15. 

RI technology is one of the most investigated methods for glucose monitoring since having access to a sample of glucose means it has a high level of accuracy. However, as Table 22 shows, the technology presents certain disadvantages [86] that some groups are still trying to overcome.

## 5. Commercial Devices

The development of non-invasive devices for blood glucose measurement dates back three decades ago when the first devices were released. Some of them showed promising results, but for several reasons, they did not succeed. For example, GlucoWatch^®^ (Cygnus Inc., Redwood, CA, USA), based on reverse iontophoresis, received FDA approval and its accuracy was evaluated as satisfactory in both clinical and home trials [130,131]. Unfortunately, due to reliability and consistency issues [132,133], the device did not survive for a long time ion the market. Also, Pendra^®^ (an impedance spectroscopy device (Pendragon Medical Ltd., Zurich, Switzerland); followed a similar fate. The device received CE approval and was commercialized for a short time, but studies after its release showed its poor accuracy [134]. Meanwhile, others never even made it to the market due to lack of funding, poor accuracy or other unclear circumstances. A good example is C8 Medisensors (Raman spectroscopy; San Jose, CA, USA) that needed an additional influx of capital to finalize the design. Unfortunately, it could not raise the money and had to shut down operations in 2013 [135]. Also, the Diasensor 1000 (Biocontrol Technology Inc., Pittsburgh, PA, USA), which is still surrounded in mystery since it is not clear whether it ever worked at all or if there were other reasons for its disappearance from the market. These and other devices with a similar fate are listed in Table 23.

Currently, there are many new devices and others that are progressively improving their technology and already in the market. Some of them are listed in Table 23, along with those developed as a proof-of-concept or still under research, but with great promise due to preliminary results. It is clear to notice that many of them use a spectroscopic technique, especially NIR, while others still need access to the interstitial fluid in a minimally-invasive (MI) way. In between those two groups, probably the most remarkable is GlucoTrack (Integrity Applications, Ashdod, Israel) due to the combination of three technologies to compensate for the disadvantages of each one. Also, it is interesting to note that while some manufacturers use PEG error grid to evaluate the accuracy of their product, others still use CEG, although its recognition as a good accuracy indicator is being left behind by approval agencies and the ISO standard. As such, it is essential to read the information shown in Table 24 with caution.

Furthermore, it is also important to note that manufacturers of NI devices aim for non-continuous glucose monitoring (NCGM), while those using MI techniques are more suitable for continuous monitoring. One reason for this could be that NI devices, especially those based on optical or vibrational spectroscopy, need measurements to be taken in a controlled environment, free of mechanical vibrations and other sources of interference like light and temperature changes. MI devices are not sensitive to such conditions, and so they can be used as continuous monitoring devices.

## 6. Sensors under Research

Various research institutions and universities are also advocated to study and develop new technologies for non-invasive detection of glucose levels. Some of them work on just one particular aspect of the technology, such as the Ulsan National Institute of Korea (UNIST) developing soft lenses for smart contact lenses including glucose detection. Others, such as Infratec and the MIT that have developed ‘Proof of concept’ designs, without any further plans for commercialization, represent potential new avenues that other groups or developers could further investigate. It is also interesting to note that although some groups explore further techniques in NIR/MIR spectroscopy, others have started looking into other alternatives. In this line, it is worth to mention the research performed by Siegel et al. at Caltech [117,158,159]. They have been doing steady progress throughout the years using waveguides, in the millimeter band, and obtaining promising results with a good correlation with the standard invasive method in rats and humans [158,159]. Also, the research taking place at the University of Western Ontario [160] is unique, although purely theoretical at this stage. They have started delving into the possibility of using nanoparticles under the principle of fluorescence-resonance energy-transfer to achieve a high degree of specificity in order to detect glucose levels in tears. 

Table 25 details these and some other current research developments that the authors consider have very favorable prospects of becoming commercial products, although some of them not in the near future.

## 7. Glucose Monitoring Informatics (GMI)

The development of more sensitive sensors using different technologies is accompanied by intensive research and development of different algorithms in order to enhance the accuracy and reliability of the sensors, to improve the readability of the data, and to compensate for the disturbance from several environmental and physiological processes, including blood perfusion, tissue scattering, sweating, and temperature changes [175]. These algorithms are also widely used to enable the development of closed-loop systems for automatic pumping of insulin in diabetic patients. Hence, algorithms can be classified into two groups. First, corrective algorithms, which aim at improving the quality of the signal itself by removing distortion due to noise and minimization of other systematic differences. The second group includes the so-called predictive algorithms, which estimate future glucose levels or enhance the current measurement based on a collection of different data sets.

### 7.1. Corrective Algorithms

There are two basic types of corrective algorithms: De-noising algorithms which eliminate or filter out noise, and enhancing algorithms which, as implied by the name, aim at improving the quality of the received signals. 

#### 7.1.1. De-Noising

As the name implies, de-noising algorithms aim at reducing the noise level of the received signal. There are several algorithms for noise removal, many of them are already used in many other fields. However, for noise removal from glucose signals, one of the best well-known techniques is the Kalman filtering. It is a linear-estimation technique which obtains a maximum likelihood estimate of the actual glucose level by evaluating the probability that the change in glucose level is due to noise, or to an actual change in glucose [176]. The technique has the advantage of being recursive, meaning that new measurements can be performed as new data arrives. It performs well in the presence of Gaussian noise by minimizing the mean square error of the estimated value. Another technique, proposed by Mahmoudi et al., is the multistep filtering comprising three stages to obtain glucose readings with reasonable accuracy. However, only the first two steps focus on noise removal. Initially, the algorithm uses a weighted local polynomial to assess glucose changes not related to physiological phenomena. Then, in the second stage, the noisy parts of the signal are detected and selectively smoothed using an exponential weighted moving average (EWMA) filter [177]. 

#### 7.1.2. Enhancement

Enhancement algorithms aim at minimizing the systematic differences in the data due to variations in the calibration and sensitivity of the glucose sensor. Some of the adopted techniques include the stochastic deconvolution-based re-calibration algorithm [178], dynamic global model (GM) [179], and the LMI-based approach [180]. Furthermore, while each of them has its own characteristics and suitability for specific scenarios, in CGM monitoring, they all need an update of the calibration parameters of the sensing system using reference data from SMBG devices, meaning that there is a certain degree of invasiveness associated with these algorithms.

### 7.2. Predictive Algorithms

Also known as glucose predictors, these algorithms analyze and weigh data of previous glucose values or other physiological parameters in order to improve the accuracy of future glucose readings. Two basic approaches exist in this category. 

#### 7.2.1. Past-Data Training Approach

Since some of the NI/MI technologies are suitable for continuous glucose monitoring, algorithms based on time-series analysis are a good approach for the prediction problem. Among them, first-order autoregressive model (ARM), first-order polynomial model, and 10th order data-driven ARM have provided reliable predictions for up to 30 minutes in advance in some experiments [181,182]. Other methods use neural network monitoring (NNM), in which the training period can take several hours. However, once it finishes, the model can run in real-time and provide good accuracy. The only issue is that NNM is not effective when detecting sudden changes in glucose concentration, meaning that it is not suitable for diabetes type-1 patients [183].

#### 7.2.2. Multi-Sensor Approach

In this approach, the signals from glucose sensors are combined with data from other sensors measuring temperature, movement, humidity and blood perfusion, among others, as well as information about carbohydrate intake, type of insulin, insulin doses, stress and amount of exercise. The combined signals provide an estimate of the glucose value through different mathematical models requiring calibration to some extent. Moreover, depending on the type of glucose sensor, and the nature of the other signals, some algorithms are more suitable than others. For example, if the data set is linear, algorithms such as principal component analysis (PCA), partial least squares (PLS) and least absolute shrinkage (LASSO) are efficient [184]. However, if the data has a non-linear nature, such as Raman spectroscopy, non-linear algorithms, such as logistic regression (LR) and support vector machines (SVM), are more convenient [185]. Besides, machine learning and artificial neural networks have been receiving much attention since they can describe problems that cannot be handled by highly non-linear algorithms or in situations in which variables evolve or mutate from the initial conditions [183]. As a result, neural networks have been extensively used in CGM technology to predict subsequent blood glucose values by using SMBG data as input, in a similar way to using the enhancement algorithms [186,187,188].

### 7.3. Closed-Loop Therapy

As explained in the previous section, using prediction algorithms makes it possible to forecast future levels of glucose in short and long terms (from less than a couple of minutes to several minutes), meaning that states such as hypoglycaemia and hyperglycaemia can be predicted and safely managed. This approach opens the door to the so-called ‘artificial pancreas’ or ‘closed-loop’ systems, which pump a certain amount of insulin automatically into the patient according to the forecasted value of glucose level in order to avoid critical glycaemic states [185]. Those systems are highly useful for type-1 patients [178].

## 8. Discussion and Future Trend

### 8.1. Past and Present of Non-Invasive Glucose Detection

John L. Smith in his book “*The Pursuit of Noninvasive Glucose: Hunting the Deceitful Turkey*” [189], accurately summarizes the past and current developments in non-invasive glucose monitoring. Many devices soon after coming out into the market, such as the widely advertised GlucoWatch and Pendra, were quickly discarded mainly due to inaccuracy and usability issues. In the same line, projects such as the contact lenses that were being developed by Google and Novartis caused great expectation, but once again, no further progress continued due to difficulties in detecting glucose accurately from the tears. As a result, these and many other unsuccessful outcomes have left many wondering if it is possible to measure glucose in a way that doesn’t need any blood drawn out of the body. Fortunately, it seems like the answer to that hope is quite optimistic.

During the last decade, much progress has happened not only from the technological point of view but also, from the regulatory field. Standards and approval agencies are seriously taking into account the existence of non-invasive glucose devices and are already setting the guidelines for their approval and use in many countries. For manufacturers and developers, this might look cumbersome as they have to comply with stricter evaluation criteria. However, this also indicates that the technology that seemed to belong only to the realm of science fiction is now in the view of becoming achievable, as proven by some devices currently available in the market and others that are close to commercialization. All of them based on different technologies that have been evolving over the years.

### 8.2. Current Research on Non-Invasive (NI) Glucose Monitoring

**Figure 16:**
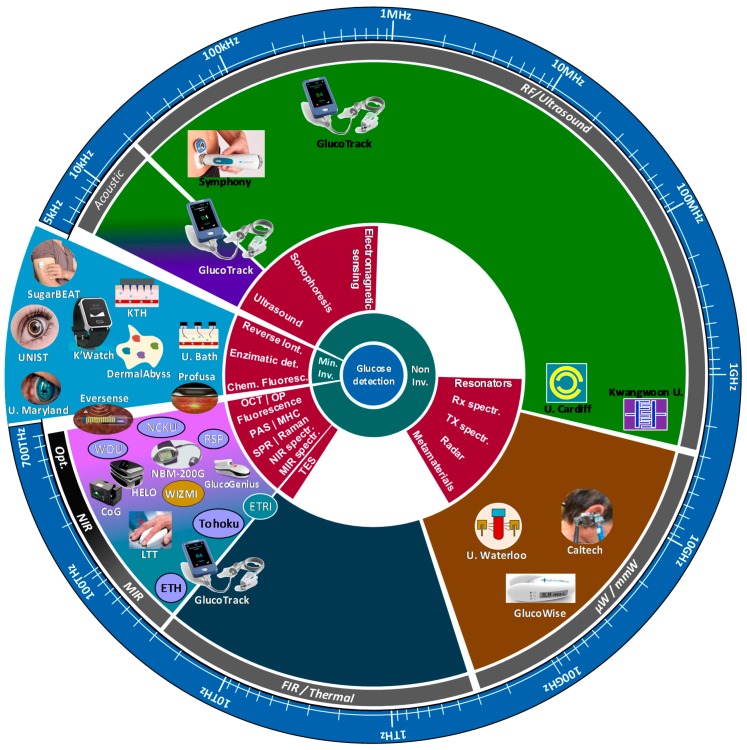
Chart of the current minimally-invasive and non-invasive technologies and devices for glucose and their location in the frequency spectrum.

The technologies described in the present review have been investigated and developed at different points in time. However, as shown in Figure 16, most of the research and effort has been put on the nanometer band, corresponding to MIR, NIR (mostly) and optical, many of which have provided optimistic results. The main reason for this is that the glucose molecule has many interesting qualities, including clear absorption lines, acceptable skin depth penetration and particular vibration modes due to atomic bonds between carbon, oxygen and hydrogen, which makes the glucose molecule easily identifiable with current equipment. 

Unfortunately, the problem is that many other molecules and fluids possess similar characteristics, posing interference, selectivity and sensitivity issues that have been the cause of failure in many previous devices. As a result, many research groups and developers have been studying other frequencies in the spectrum that might help to overcome some of the issues previously mentioned, especially in the ultrasound, low-RF and microwave regions, many of which have already started providing promising results. Figure 16 shows three large regions that still need further research, the mid-band of the RF spectrum, between 1 MHz and 1 GHz, the upper millimeter band and most of the FIR region, between 100 GHz and 30 THz. In the case of the mid-RF band, most of the research is limited only to measurements in-vitro, but results such as those obtained by Kim et al. [3], are encouraging, especially knowing that penetration into the skin and tissue would not represent an obstacle due to the long wavelength. The only foreseeable obstacle is the presence of many applications, including communications, radar, TV, and radio, among others, which heavily use the RF band, and so they represent a potential interference issue. As for the upper-mmW/FIR band, the main issues are the strong water absorption, which renders most measurements useless due to low-depth penetration; and the lack of sources capable of producing meaningful amounts of energy that can penetrate the tissue for non-invasive analysis. However, recent developments in Quantum-cascade-lasers (QCLs) might open a window into studying the glucose molecule in such region but the cost and size associated make any significant advancement unfeasible for a while.

All in all, it is quite clear that industry and research institutions have favored optical and near-infrared techniques for developing non-invasive glucose detection technology. However, a question we should ask is how far can those research efforts go without relying on other technologies to complement their techniques? It is clear that NIR and optical techniques have serious problems with interference, movement sensitivity, and attenuation, among others. Hence, several other groups have been focusing on using other technologies based on ultrasound, RF, and non-electromagnetic approaches to overcome the aforementioned limitations. However, each of those techniques also present their limitations. As such, we think the brighter future for non-invasive glucose detection might rely on the combination of several techniques in one single device in order to analyze the characteristics of glucose from different angles, and with that, get meaningful data that can lead towards the development of a truly accurate and reliable non-invasive glucose monitoring device.

### 8.3. Current Research on Minimally-Invasive (MI) Glucose Monitoring

The issue of whether MI devices are part of the non-invasive technology or not is still under discussion. On one side, MI ticks the marks of comfortability, usability and accuracy, which are the main points for a device to become accepted in the market. On the other hand, the issue of still needing access to internal fluids, posing potential infection risks, puts several MI devices on the group of technologies to be forgotten once NI becomes fully developed and matured. Especially, since it is quite clear, from our point of view, that the tendency of personal diagnostic devices is leaning towards wearable technology demanding for safe, easy-to-use, lightweight and cost-effective items.

Nevertheless, the research for glucose detection using MI techniques continues, and it has even provided several promising alternative approaches, especially those based on reverse iontophoresis and fluorescence. In the case of reverse iontophoresis, the devices are still based on the fundamental principle of glucose oxidation via an enzyme (ex. GoX), which means that they still need access to a certain amount of glucose, making them part of the invasive-device category. However, with new miniaturization processes, several research groups are working with microneedles to access the interstitial fluid without any sensation of discomfort or pain, and so, products such as the K’Watch^®^ (PKVitality, Paris, France) are close to commercialization.

Fluorescence has also been going through intensive research, especially towards the development of glucose monitoring devices underneath the skin, such as the Eversense^®^ from Senseonics (Germantown, MD, USA) that is already available, and a new device under development by Profusa (San Francisco, CA, USA), currently under development. Both devices are quite promising since the measurement procedure is entirely non-invasive via fluorescent light. However, the fact that both devices need to be under the skin means that there is an initial invasive procedure involved. Also, both of them have limited time life (180 days in the case of Eversense^®^), meaning that there is still additional research to be done to prolong the sensor’s life, especially regarding the development of new types of coating to delay or inhibit the recognition of foreign material.

In addition, contact lenses have also been under study using different technologies, including optical polarimetry [190,191], electrochemical sensing [192,193], fluorescence [171], and other photonic-based approaches [194,195,196]. However, glucose measurement on tears has proven to have several difficulties, preventing even giants such as Google and Novartis from achieving further progress, given issues connected to the small amount of glucose in tears, lag time, sample collection and mechanical issues. Nevertheless, given the large potential market and the life-changing factor for many people with diabetes around the world, several groups are still working on a solution. The development is still at an early stage, but if successful, we are certain that contact lenses would be a serious contender to non-invasive devices given their potential use for tracking many other physiological parameters, and the assessment and treatment of certain medical conditions [197], all in one single device.

### 8.4. Considerations for Future Developments in NI and MI Glucose Monitoring

There are still key challenges to be addressed before having an entirely reliable non-invasive device for glucose measurement, despite seeing many groups and companies getting closer day by day to this point. For example, many of the current devices already comply with the guidelines given by ISO 15197:2003, which are still used as the main approval guideline in many countries. Moreover, even though the bar has been lifted with stricter requirements in ISO 15197:2013, adopted by the European Union in 2016, and the always demanding FDA requirements in the USA, the research is still promising, and highly competitive given the vast potential markets around the world.

Current standards and regulations for point-of-care and self-assessment of glucose address the performance of SMBG and CGM devices only. Also, throughout the years, standards have evolved to the point, where evaluation indicators of reliability and accuracy are now very tight. Hence, any new approach will be required to provide an equivalent performance or improvement over invasive technologies, or offer new insights on diabetes management and safe glucose levels and change rates. All this poses a great challenge for NI and MI to become genuinely accepted, as their performance will have to become comparable or even better than that of CGM and SMBG. However, as technologies improve, this will become feasible, and so it will mark the arrival of standards that especially focus on NI/MI detection. Guidelines on accuracy and reliability of the new standards might be the same as those regulating CGM and SMBG technologies, but new guidelines regulating the unique characteristics of NI/MI technologies will also be necessary, especially those associated with the safety of the patient when exposed to a new type of radiation. For example, we consider that some guidelines will need to define: (i) the type of radiation that could be applied, i.e., non-ionizing radiation; (ii) the maximum power of the signal; (iii) parts of the body that are safe to scan; and (iv) interference issues with other medical and communication devices.

Also, during the last decade, we have seen an exponential growth of wearable technology, i.e., devices that merge with regular daily clothing, in the form of wrist watches, finger rings, arm bands, chest bands, earrings, among others. With them, it is now possible to collect a diverse range of physiological parameters. As a result, people now can count their steps, monitor their heart rate, measure their blood pressure, and even track their daily calorie intake, among other parameters. This new revolution represents an excellent opportunity for the fields of big data and machine learning, allowing the anonymous collection and processing of extensive data sets of many endogenous and exogenous variables. Such approach will permit the development of (i) more accurate prediction models that can enhance the accuracy and reliability of non-invasive glucose detection; (ii) enhancement of current signal-processing algorithms; and (iii) new research insights of the different internal and external factors leading to hypoglycaemic and hyperglycaemic events.

Added to this progress, mathematical and statistical algorithms help to improve the accuracy of NI/MI technologies. However, their main limitation, especially in the case of predictive algorithms, is that apart from having been exclusively designed to improve the performance of CGM devices, they depend heavily on accurate and reliable glucose measurements for calibration. Hence, the usability of NI/MI devices, in closed-loop systems, is quite limited, since they are still on a premature state of development, compared to CGM and SMBG technologies. Nevertheless, they have already achieved an acceptable degree for trend estimation which makes them suitable as complementary tools for the prevention of critical events. However, once they reach higher maturity, and algorithms are better adapted to the signal characteristics of NI and MI, it will be possible to sense glucose concentrations at higher rates and obtain more significant and more varied sets of data, in conjunction with wearable technology, leading to a change in the rules for insulin dosing in closed-loop therapy, i.e., dosing frequency and insulin amount per dose.

Finally, needless to say is the fact that NI devices will unlikely replace or at least achieve the same accuracy level as the standard laboratory equipment, discussed in Section 2.1, given their high degree of reliability and specificity to detect glucose over a wide range of concentrations. As such, laboratory equipment will continue being the central tool for accurate glucose readings and the primary gold-standard for test and calibration of home-monitoring devices. On the other hand, we believe that the replacement of CGM and SMBG devices with a non-invasive option is achievable and the primary target for all researchers and developers at this point in time. However, reaching such point will require not only the development of more sensitive and specific sensors that can detect glucose under different conditions, but also the development of new data-analysis and algorithm techniques as interference, attenuation, and noise will always cloud the glucose signal. As a result, it is also crucial to develop more efficient algorithms, statistical analysis techniques and mathematical models that can allow an accurate differentiation of the glucose molecule.

## 9. Conclusions

The present review has provided a technical view of the current technologies and devices aimed at monitoring glucose levels without the need of blood samples. It has also detailed the techniques and tools to evaluate their accuracy, the technologies used in standard equipment and devices, and the regions in the electromagnetic spectrum in which most of the research is taking place. Combining all these sections, we have tried to show the way in which all the current aspects, connected to glucose detection, model the technical evolution and development of future devices that can monitor glucose concentrations non-invasively.

Without putting any demerit on all the research and progress currently being undertaken, it is essential to ask ourselves the level of accuracy that an NI glucose detection device should have before considering it “good enough”. Without any doubt, the issue of getting an accurate reading of the glucose level in our body has proven to be quite challenging, but what if we demand too much on something that does not need to be that precise? For many critical diabetic patients, including those with diabetes type 1, whose glucose levels are subject to sudden changes, accurate and continuous sensing is a matter of life or death. However, it should also be acknowledged the existence of many other millions of people whose glucose levels do not need continuous monitoring or accurate reading since they do not suffer from unexpected glucose variations. Many even only need to know if their glucose level is within an acceptable range or not, such as those with diabetes type 2, and go for a more comprehensive test only if some abnormality is detected. Following this idea is what the developers of the HELO Extense (NIR spectroscopy; World Global Network, Miami, FL, USA) [144], and the DermalAbyss (chemical fluorescence; MIT, Cambridge, MA, USA) [169], have been trying to achieve using a color based indicator for glucose concentration. 

Will this be enough for finally hunting the deceitful turkey? For many, the answer will probably be affirmative, but for many others needing continuous monitoring with exact results, the good news will take longer to arrive, as a new regulatory framework will also need to keep the pace with technological development. Low sensitivity, low specificity and interference keep presenting themselves as the main obstacles due to several limitations in the hardware and software currently under use. However, with the appearance of new technologies and the continuous improvement of the old ones, we are convinced that the appearance of the genuinely non-invasive glucose is just a matter of time.

## Figures and Tables

**Table 1:** Representative equipment used for accurate glucose measurement in the laboratory.

Method	Equipment	Intended Use	Sample Type	Range	Ref.
**Enzymatic**	YSI 2700	Laboratory Point-of-care	Blood Plasma Serum CSF	0–2500 mg/dL	[14]
	YSI 2950D	Laboratory		5–2500 mg/dL	[15]
	Biosen C-Line/S-Line	Laboratory Point-of-care	Blood Plasma Serum	9–900 mg/dL	[16]
**Hexokinase**	Beckman Coulter DxC 800	Laboratory	Plasma Serum Urine CSF	5–700 mg/dL	[17]
	Abbott ARCHITECT c8000/c16000			1–800 mg/dL	[18]
	Hitachi 917			2–750 mg/dL	[18]
	Cobas c 701/702			2–750 mg/dL	[19]

**Table 2:** Comparison between laboratory and self-monitoring techniques.

Characteristics	Laboratory	Self-Monitoring
**Accuracy**	Very good	Good
**Sensitivity**	Very good	Good
**Measurement time**	Long	Quick
**Trained laboratory personnel**	Yes	No
**Sample type**	Blood, serum, plasma, urine	Blood, ISF
**Blood extraction method**	Invasive	Invasive

**Table 3:** Standard error grids for clinical accuracy assessment of glucose detection.

	Clarke Error Grid [40]	Parkes Error Grid Type 1 Diabetes [41]	Parkes Error Grid Type 2 Diabetes [41]	Surveillance Error Grid [38]
Risk zones	A to E	A to E		Green to Dark-red
A 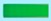 Green	Clinically correct decisions	No effect on clinical action		No risk
B 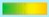 G/Y	Clinically uncritical decisions	Altered clinical action or little or no effect on clinical outcome		Mild risk
C 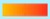 Y/R	Overcorrections that could lead to a poor outcome	Altered clinical action: likely to affect clinical outcome		Moderate risk
D 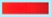 Red	Dangerous failure to detect and treat	Altered clinical action: potential significant medical risk		High risk
E 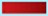 Dark Red	Erroneous treatment	Altered clinical action: potential dangerous consequences.		Extreme risk

**Table 4:** Guidelines for approval of glucose monitoring devices in some countries.

Agency	Country	Guideline/Standard	Release Year	Device Type	Glucose Concentration	Criteria
Food & Drug Administration (FDA)	USA [47,48]	UCM 380325	2016	BGMS	≥75 mg/dL	95% within ±12% 98% within ±15%
					<75 md/dL	95% within ±12 mg/dL 98% within ±15 mg/dL
		UCM 380327		SMBG	Entire range	95% within ±15% 99% within ±20%
European Medicines Agency (EMA)	EU [49]	EN ISO 15197	2015	BGMS/SMBG	≥100 mg/dL	95% within ±15%
Health Canada	Canada [50]	ISO 15197	2013		<100 mg/dL	95% within ±15 mg/dL
Agência Nacional de Vigilância Sanitária (ANVISA)	Brazil [51]					
China Food & Drug Administration (CFDA)	China [52]					
Pharmaceuticals and Medical Devices Agency (PMDA)	Japan [53,54]				Entire range (Type 1 Diabetes)	99% within Zones A & B of Parkes Error Grid
Therapeutic Goods Administration TGA	Australia [55,56,57]					

**Table 5:** Advantages and disadvantages of Surface plasmon resonance.

Bioimpedance Spectroscopy	
*Advantages*	*Disadvantages*
Highly sensitive to small changes of blood glucose concentration. No need for statistical calibration models due to its conventional electrical model nature.	Sensitive to motion. Long calibration process Sensitive to sweat and temperature. Bulky in size

**Table 6:** Advantages and disadvantages of fluorescence technology.

Fluorescence	
*Advantages*	*Disadvantages*
Very sensitive to glucose concentrations as small as 25 μM, allowing even single-molecule detection. High specificity due to unique optical properties of molecules. It can measure analyte concentration in terms of fluorescence intensity and decay times. Immune to light scattering	Susceptible to interference due to pH changes and oxygen levels. Potential toxicity issues due to foreign material in biological media. Short lifespan of the fluorophore. Limitations associated with photostability and loss of recognition capability. Biocompatibility issues due to local tissue trauma. Susceptible to autofluorescence

**Table 7:** Advantages and disadvantages of Optical Polarimetry.

Optical Polarimetry	
*Advantages*	*Disadvantages*
Very high resolution. Optical components can be easily miniaturized.	Sensitive to temperature changes and motion. Sensitive to interference from other optically active compounds. Lag time could be up to 30 min.

**Table 8:** Advantages and disadvantages of Optical Coherence Tomography.

Optical Coherence Tomography	
*Advantages*	*Disadvantages*
Very high resolution. High signal to noise ratio. High penetration depth. Not susceptible to blood pressure, heart rate and hematocrit.	Sensitive to temperature changes on the skin and motion. Susceptible to tissue inhomogeneity.

**Table 9:** Advantages and disadvantages of NIR spectroscopy.

NIR Spectroscopy	
*Advantages*	*Disadvantages*
Water transparent in the NIR band Relatively low-cost materials needed. The signal intensity is directly proportional to the concentration of the analyte. Minimum sample preparation required. Method also works in presence of interfering substances, such as glass or plastic containers.	Heterogeneous distributions of glucose can give false readings. Glucose concentrations are too low for accurate detection. High scattering level Problems of selectivity for determination of glucose.

**Table 10:** Advantages and disadvantages of NIR spectroscopy.

MIR Spectroscopy	
*Advantages*	*Disadvantages*
Low scattering. The absorption bands are more specific and better delineated. The absorption of MIR radiation by glucose is stronger than in the NIR band. Glucose can absorb specific MIR wavelengths, thus its concentration can be measured with more accuracy.	Penetration depth is just a few micrometers. Only reflection is feasible due to poor penetration. Strong water absorption. Expensive equipment.

**Table 11:** Advantages and disadvantages of Raman spectroscopy.

Raman Spectroscopy	
*Advantages*	*Disadvantages*
Less sensitive to temperature changes. Minimally sensitive to water. Suitable on any surface since it measures scattered light, including opaque substrates. High specificity.	Prone to interference from other molecules such as haemoglobin. Unstable laser wavelength and intensity. Long collection time. Susceptible to noise interference (low signal to noise ratio), fluorescence and turbidity.

**Table 12:** Advantages and disadvantages of FIR spectroscopy.

FIR Spectroscopy	
*Advantages*	*Disadvantages*
Less scattering than NIR and MIR	Strong water absorption makes extremely difficult the identification of other molecules in the sample.

**Table 13:** Advantages and disadvantages of TOF and THz-TDS.

TOF/THz-TDS	
*Advantages*	*Disadvantages*
Immune to background noise. Study of a broad frequency range with a single ultrashort pulse. Complex permittivity measurement with a single scan.	Long measurement time Low spatial and depth resolution

**Table 14:** Advantages and disadvantages of thermal emission spectroscopy.

Sonophoresis	
*Advantages*	*Disadvantages*
It is a passive technique. No risk of damaging tissue Good selectivity given the well-defined spectra of glucose at 9.4 μm. No calibration required.	Sensitive to variations of temperature and motion. Intensity of radiation susceptible to the thickness of the tissue. It might not be suitable for detecting sudden changes of glucose.

**Table 15:** Advantages and disadvantages of MHC.

Metabolic Heat Conformation	
*Advantages*	*Disadvantages*
Physiological parameters are relatively easy to measure using well established technologies.	Susceptible to interference by environmental conditions, including temperature [108] Sensitive to sweat.

**Table 16:** Advantages and disadvantages of photoacoustic spectroscopy.

Photoacoustic Spectroscopy	
*Advantages*	*Disadvantages*
Relatively simple method. Immune to water distortion. Not susceptible to NaCl, cholesterol, and albumin. PA signal is not influenced by scattering particles.	Susceptible to changes of temperature, pulsation, motion and surrounding acoustic noise. Low signal-to-noise ratio. Long integration time.

**Table 17:** Advantages and disadvantages of millimeter and microwave sensing.

Millimeter and Microwave Sensing	
*Advantages*	*Disadvantages*
Signal penetration is deep enough to reach tissues containing sufficient glucose. No risk of ionization. Sensitive to small changes of glucose concentration.	Susceptible to biological differences in blood. Sensitive to variations of physiological parameters, including breathing, sweating level and cardiac activity [123]. Poor selectivity.

**Table 18:** Advantages and disadvantages of electromagnetic sensing technology.

Electromagnetic Sensing	
*Advantages*	*Disadvantages*
Using a single frequency, specific to the analyte, minimizes interference caused by other media. There is no ionization risk.	Highly sensitive to temperature.

**Table 19:** Advantages and disadvantages of bioimpedance spectroscopy.

Bioimpedance Spectroscopy	
*Advantages*	*Disadvantages*
Relatively inexpensive. Easy measurement on the skin.	Sensitive to variations of temperature and motion. Sensitive to sweat and to water content. Affected by physiological conditions affecting the cell membrane.

**Table 20:** Advantages and disadvantages of Ultrasound technology.

Ultrasound	
*Advantages*	*Disadvantages*
It can travel long distances below the skin or tissue. Immune to skin color variation.	Susceptible to ambient temperature.

**Table 21:** Advantages and disadvantages of sonophoresis.

Sonophoresis	
*Advantages*	*Disadvantages*
No adverse effects on the skin. Glucose measurement is based on the well known enzymatic method. Better control on the amount of glucose that can be extracted for analysis.	Susceptible to temperature variations. Interference from other compounds and pressure changes.

**Table 22:** Advantages and disadvantages of Reverse iontophoresis.

Reverse Iontophoresis	
*Advantages*	*Disadvantages*
Electrodes are not difficult to manufacture and be applied to the skin with minimum training. Good correlation between glucose level in the ISF and in the blood under stable conditions. Glucose measurement is based on the well known enzymatic method.	Skin irritation due to the pass of current. Susceptible to sweating. Rapid changes of glucose concentration cannot be detected accurately.

**Table 23:** List of commercial devices for non-invasive glucose monitoring never released or withdrawn.

Device	Technology
GlucoWatch	Reverse iontophoresis
GluCall	Reverse iontophoresis
Pendra	Impedance spectroscopy
Glucoband	Impedance spectroscopy
Hitachi Ltd.	Metabolic heat conformation
Aprise	Photoacoustic spectroscopy
C8 Medisensors	Raman spectroscopy
Diasensor 1000	NIR spectroscopy
TouchTrack Pro	NIR spectroscopy
GluControl	NIR spectroscopy

**Table 24:** Comparison table of minimally-invasive and non-invasive glucose monitoring devices currently available or close to release.

Device	Technology	Target	Type	Accuracy	Status	Ref.
Combo Glucometer (*Cnoga Medical*)	NIR spectroscopy (combination of four LEDs and four sensors to analyse absorption and scattering pattern) λ: 625 nm, 740 nm, 850 nm, 940 nm	Finger	NI NCGM	PEG Zone A: 96.6% Zone B: 3.4% MARD: 14.4%	Available	[136,137,138,139]
NBM-200G* (*OrSense*)	NIR spectroscopy (Occlusion spectroscopy) λ: 610 nm, 810 nm	Finger	NI Point-of-care	CEG Zone A: 69.7% Zone B: 25.7%	Dropped	[140,141,142,143]
HELO Extense (*World Global Network*)	NIR spectroscopy	Finger	NI NCGM	N/A	Available	[144]
GlucoTrack (*Integrity Applications*)	Combination of: Ultrasound Thermal Electromagnetic sensing	Ear lobe	NI NCGM	PEG Zone A: 62.4% Zone B: 37.6% MARD: 19.7%	Available	[127,145,146]
GlucoWise (*MediWise*)	mm-Wave Transmission spectroscopy f: 60 GHz	Hand	NI NCGM	N/A	Under develop-ment	[118,147]
SugarBEAT (*Nemaura Medical*)	Reverse iontophoresis	Upper arm	MI CGM	MARD: 13.76%	Waiting for CE approval	[148]
Symphony (*Echo Therapeutics*)	Sonophoresis	Skin	MI CGM	CEG Zone A: 81.7% Zone B: 18.3% MARD: 12.3%	Unknown	[149]
Wizmi^TM^ (*Wear2b Ltd*)	NIR spectroscopy	Arm wrist	NI CGM	CEG Zone A: 93% Zone B: 7% MARD: 7.23%	Proof of concept	[150]
LTT (*Light Touch technology*)	MIR spectroscopy/Optical Parametric Oscillation λ: 6–9 μm	Finger	NI NCGM	N/A	Under develop-ment	[151]
K’Watch (*PKvitality*)	Enzymatic detection/microneedles	Arm wrist	MI CGM	N/A	Pre-clinical tests	[11,152,153]
Eversense^®^ (*Senseonics*)	Fluorescence	Upper arm	MI CGM	MARD: 14.8%	Available	[154,155,156]
Health-Care Computer	Metabolic heat conformation λ: 660 nm, 760 nm, 850 nm, 940 nm	Finger	NI NCGM	87%	Available	[107,157]
GlucoGenius				N/A	Unknown	
GlucoDiary						
G2 Mobile (*Eser Digital*)						

* Although the device has not been further developed, still occlusion spectroscopy is seriously considered as a feasible technology for glucose monitoring. PEG: Parkes Error Grid, CEG: Clarke Error Grid, N/A: Not available.

**Table 25:** List of current research on NI and MI technologies for glucose detection.

Institution	Technology	Comments	Target	Ref.
Polytechnic University of Catalunya	NIR spectroscopy Photoplethysmo-graphy	Principle: relationship between PPG waveform and glucose levels. No calibration needed Linear response even in hypoglycemia and hyperglycemia	Finger	[161]
Karunya University	NIR spectroscopy Photoplethysmo-graphy	Blood viscosity, breathing, emotional state and autonomous nervous system are linked to glucose levels. Analysis done with machine learning.	Forearm & finger	[162]
Tohoku University	MIR spectroscopy Trapezoidal multireflection	Suitable for areas without thick skin layer. Tuned at 8658 nm. Sensitive to contact pressure	Oral mucosa Inner lips	[163]
ETH Zurich	MIR spectroscopy Photoacoustic detection	It uses Quantum Cascade lasers (QCLs). Wavelengths: 8.47–10 μm	Forearm	[111]
RSP Systems	Raman spectroscopy	Glucose sensing at a critical depth in the skin. Accuracy affected by time-lag λ: 830 nm	Hand palm	[164]
University Western Ontario (WOU)	Fluorescence Resonance Energy Transfer (FRET)	Spectral determination based on competition reaction between fluorophore’s donor and acceptor	- - -	[160]
Electronics and Telecomm. Research Inst. of Korea (ETRI)	Photoacoustic spectroscopy	Insensitive to skin secretions. Acoustic signal: 47 kHz λ: 8–10.4 μm	Fingertip	[112]
National Cheng Kung University (NCKU)	Optical Coherence Tomography	It senses optical rotation angle (γ) and depolarization index (Δ) using Mueller model. Increase of glucose, increases γ and decreases Δ.	Fingertip	[165]
Caltech	Millimeter-wave Transmission	Based on waveguides and patch antennas *f*: 15–25 GHz, 16–36 GHz	Ear lobe	[117,159]
University of Waterloo	Millimeter-wave transmission & reflection	Based on Google’s Soli system and Forest classifier. Sensitive to differences in blood. *f*: 60 GHz	- - -	[116]
University of Erlangen-Nuremberg	Millimeter-wave transmission & reflection	Changes in the glucose level are correlated with variations in the amplitude and phase of the transmitted and reflected waves. Glucose characterization between 0.2 and 40 GHz *f*: 5–8.6 GHz (patch & slot antennas)	- - -	[119,166]
Cardiff University	Microwave Split-ring resonance	Glucose level change shifts resonant frequency. Up to 17.5 mm depth penetration.	Abdomen	[120,167]
University of Bath	Reverse iontophoresis	Based on the electro-osmotic flow principle. ISF extracted through hair follicles Independent from skin characteristics variance Some skin irritation associated	Skin	[168]
MIT–DermalAbyss	Chemical fluorescence	Tattoo injected in the dermis. No power needed to operate Tattoo changes color depending on the concentration of glucose	Skin	[169]
Ulsan National Inst. of Science and Technology (UNIST)	Contact lenses–Enzymatic detection	Measures the level of glucose in tears Electrodes embedded in the contact lens. Lag time between 10 and 30 min Interference from other electroactive species	Tears	[170]
University of Maryland	Contact lenses-fluorescence	Based on a glucose-silicone hydrogel. Decrease of fluorescence with increase of glucose It works with fluorophore Quin-C18 Long storage seems not to affect lens’ response	Tears	[171]
KTH Royal Inst. of Technology	Microneedle-Enzymatic detection	Measurement taken within the dermis. Based on passive fluid extraction. Microneedle length: 700 μm	Forearm	[172]
Profusa, Inc.	Fluorescence	Placed under the skin. Based on fluorescence of anionic dyes. Flexible fiber, 3–5 mm long, 500 μm diam. Fluorescent light detected with external sensor.	- - -	[173,174]

## References

[1] World Health Organization (WHO) Diabetes. http://www.who.int/news-room/fact-sheets/detail/diabetes.

[2] Healthline The Effects of Low Blood Sugar on Your Body. https://www.healthline.com/health/low-blood-sugar-effects-on-body#6.

[3] Clark L.C., Lyons C. (1962). Electrode systems for continuous monitoring in cardiovascular surgery. Ann. N. Y. Acad. Sci..

[4] So C.-F., Choi K.-S., Wong T.K.S., Chung J.W.Y. (2012). Recent advances in noninvasive glucose monitoring. Med. Dev. (Auckl.).

[5] Uwadaira Y., Ikehata A., Bagchi D., Nair S. (2018). Noninvasive Blood Glucose Measurement. Nutritional and Therapeutic Interventions for Diabetes and Metabolic Syndrome.

[6] Cho N.H., Shaw J.E., Karuranga S., Huang Y., da Rocha Fernandes J.D., Ohlrogge A.W., Malanda B. (2018). IDF Diabetes Atlas: Global estimates of diabetes prevalence for 2017 and projections for 2045. Diabetes Res. Clin. Pract..

[7] Lin J., Thompson T.J., Cheng Y.J., Zhuo X., Zhang P., Gregg E., Rolka D.B. (2018). Projection of the future diabetes burden in the United States through 2060. Popul. Health Metr..

[8] Tura A., Sbrignadello S., Cianciavicchia D., Pacini G., Ravazzani P. (2010). A Low Frequency Electromagnetic Sensor for Indirect Measurement of Glucose Concentration: In Vitro Experiments in Different Conductive Solutions. Sensors.

[9] Chen C., Zhao X.-L., Li Z.-H., Zhu Z.-G., Qian S.-H., Flewitt A.J. (2017). Current and Emerging Technology for Continuous Glucose Monitoring. Sensors.

[10] Lin T., Gal A., Mayzel Y., Horman K., Bahartan K. (2017). Non-invasive Glucose Monitoring: A Review of Challenges and Recent Advances. Curr. Trends Biomed. Eng. Biosci..

[11] Van Enter B.J., von Hauff E. (2018). Challenges and perspectives in continuous glucose monitoring. Chem. Commun..

[12] Khalil O.S. (1999). Spectroscopic and Clinical Aspects of Noninvasive Glucose Measurements. Clin. Chem..

[13] McMillin J.M., Walker H.K., Hall W.D., Hurst J.W. (1990). Blood Glucose. Clinical Methods: The History, Physical and Laboratory Examinations.

[14] YSI Incorporated (2000). YSI 2700 SELECT Biochemistry Analyzer User’s Manual.

[15] YSI Incorporated 2950D Biochemistry Analyzer. https://www.ysi.com/ysi-2950-biochemistry-analyzer.

[16] EKF-Diagnostic GmbH. Biosen C-Line & Biosen S-Line. https://www.ekfdiagnostics.com/res/BS%20Data%20EN%20EU%205.1-02.17.pdf.

[17] Beckman Coulter Chemistry Information Sheet. https://www.beckmancoulter.com/wsrportal/techdocs?docname=/cis/B31851/%25%25/EN_GLUH.pdf.

[18] U.S. Food & Drug Administration—FDA Review Memorandum—Quantitative Enzymatic Assay Based on Hexokinase/G-6-PDH Methodology. https://www.accessdata.fda.gov/cdrh_docs/reviews/K060383.pdf.

[19] Roche Diagnostics USA Glucose HK Gen.3. https://usdiagnostics.roche.com/products/05168791190/PARAM49/overlay.html.

[20] Delost M.E., Volsko T.A., Chatburn R.L., El-Khatib M.F. (2014). Blood Gas and Critical Care Analyte Analysis. Equipment for Respiratory Care.

[21] Burrin J.M., Price C.P. (1985). Measurement of Blood Glucose. Ann. Clin. Biochem..

[22] Liang Y.M.D., Wanderer J.M.D.M., Nichols J.H.P., Klonoff D.M.D.F., Rice M.J.M.D. (2017). Blood Gas Analyzer Accuracy of Glucose Measurements. Mayo Clin. Proc..

[23] Inoue S., Egi M., Kotani J., Morita K. (2013). Accuracy of blood-glucose measurements using glucose meters and arterial blood gas analyzers in critically ill adult patients: Systematic review. Crit. Care.

[24] Dalvi N. (2013). Glucose meter reference design. Application Note Nr. 1560.

[25] Rebel A., Rice M.A., Fahy B.G. (2012). The Accuracy of Point-of-Care Glucose Measurements. J. Diabetes Sci. Technol..

[26] Ekhlaspour L., Mondesir D., Lautsch N., Balliro C., Hillard M., Magyar K., Radocchia L.G., Esmaeili A., Sinha M., Russell S.J. (2016). Comparative Accuracy of 17 Point-of-Care Glucose Meters. J. Diabetes Sci. Technol..

[27] Chakraborty P.P., Patra S., Bhattacharjee R., Chowdhury S. (2017). Erroneously elevated glucose values due to maltose interference in mutant glucose dehydrogenase pyrroloquinolinequinone (mutant GDH-PQQ) based glucometer. BMJ Case Rep..

[28] Schultz D.G. FDA Public Health Notification: Potentially Fatal Errors with GDH-PQQ* Glucose Monitoring Technology. http://labmed.ucsf.edu/labmanual/db/resource/FDA_glucometer_warning_Aug_2009.pdf.

[29] Diabetes Australia Continuous Glucose Monitoring. https://static.diabetesaustralia.com.au/s/fileassets/diabetes-australia/e2feb45e-ebc4-4133-85e1-b514e67d24de.pdf.

[30] Wadwa R.P., Fiallo-Scharer R., VanderWel B., Messer L.H., Cobry E., Chase H.P. (2009). Continuous Glucose Monitoring in Youth with Type 1 Diabetes. Diabetes Technol. Ther..

[31] Patton S.R., Clements M.A. (2012). Continuous Glucose Monitoring Versus Self-monitoring of Blood Glucose in Children with Type 1 Diabetes- Are there Pros and Cons for Both?. US Endocrinol..

[32] Nardacci E.A., Bode B.W., Hirsch I.B. (2010). Individualizing Care for the Many. Diabetes Educ..

[33] Ward J.E.F., Stetson B.A., Mokshagundam S.P.L. (2015). Patient perspectives on self-monitoring of blood glucose: perceived recommendations, behaviors and barriers in a clinic sample of adults with type 2 diabetes. J. Diabetes Metab. Disord..

[34] Danne T., Nimri R., Battelino T., Bergenstal R.M., Close K.L., DeVries J.H., Garg S., Heinemann L., Hirsch I., Amiel S.A. (2017). International Consensus on Use of Continuous Glucose Monitoring. Diabetes Care.

[35] Reiterer F., Polterauer P., Schoemaker M., Schmelzeisen-Redecker G., Freckmann G., Heinemann L., del Re L. (2017). Significance and Reliability of MARD for the Accuracy of CGM Systems. J. Diabetes Sci. Technol..

[36] Bailey T.S. (2017). Clinical Implications of Accuracy Measurements of Continuous Glucose Sensors. Diabetes Technol. Ther..

[37] Boren S.A., Clarke W.L. (2010). Analytical and Clinical Performance of Blood Glucose Monitors. J. Diabetes Sci. Technol..

[38] Klonoff D.C., Lias C., Vigersky R., Clarke W., Parkes J.L., Sacks D.B., Kirkman M.S., Kovatchev B. (2014). The Surveillance Error Grid. J. Diabetes Sci. Technol..

[39] Klonoff D.C. (2012). The Need for Clinical Accuracy Guidelines for Blood Glucose Monitors. J. Diabetes Sci. Technol..

[40] Clarke W.L., Cox D., Gonder-Frederick L.A., Carter W., Pohl S.L. (1987). Evaluating Clinical Accuracy of Systems for Self-Monitoring of Blood Glucose. Diabetes Care.

[41] Parkes J.L., Slatin S.L., Pardo S., Ginsberg B.H. (2000). A new consensus error grid to evaluate the clinical significance of inaccuracies in the measurement of blood glucose. Diabetes Care.

[42] International Organization for Standardization (ISO). https://www.iso.org.

[43] International Organization for Standardization (ISO) (2013). ISO 15197:2013. Vitro Diagnostic Test Systems—Requirements for Blood-Glucose Monitoring Systems for Self-Testing in Managing Diabetes Mellitus.

[44] Freckmann G., Baumstark A., Jendrike N., Rittmeyer D., Pleus S., Haug C. (2017). Accuracy Evaluation of Four Blood Glucose Monitoring Systems in the Hands of Intended Users and Trained Personnel Based on ISO 15197 Requirements. Diabetes Technol. Ther..

[45] International Organization for Standardization (ISO) (2015). International Organization for Standardization (ISO). In vitro diagnostic test systems—Requirements for blood-glucose monitoring systems for self-testing in managing diabetes mellitus (ISO 15197:2013). EN ISO 15197:2015.

[46] Freckmann G., Schmid C., Baumstark A., Rutschmann M., Haug C., Heinemann L. (2015). Analytical Performance Requirements for Systems for Self-Monitoring of Blood Glucose With Focus on System Accuracy: Relevant Differences among ISO 15197:2003, ISO 15197:2013, and Current FDA Recommendations. J. Diabetes Sci. Technol..

[47] U.S. Food & Drug Administration (FDA) (2016). Blood Glucose Monitoring Test Systems for Prescription Point-of-Care Use.

[48] U.S. Food & Drug Administration (FDA) (2016). Self-Monitoring Blood Glucose Test Systems for over-the-Counter Use.

[49] European Commission In vitro Diagnostic Medical Devices. http://ec.europa.eu/growth/single-market/european-standards/harmonised-standards/iv-diagnostic-medical-devices/#Note%202.1.

[50] Government of Canada New Requirements for Medical Device Licence Applications for Lancing Devices and Blood Glucose Monitoring Systems. https://www.canada.ca/en/health-canada/services/drugs-health-products/medical-devices/activities/announcements/notice-new-requirements-medical-device-licence-applications-lancing-devices-blood-glucose-monitoring-systems.html.

[51] Agência Nacional de Vigilância Sanitária (ANVISA) (2018). Instrução Normativa Nº 24.

[52] China Food & Drug Administration (CFDA) (2016). Glucometer Registration Technical Review Guidelines.

[53] Pharmaceuticals and Medical Devices Agency (PMDA) Handling of Self-Testing Blood Glucose Meters. http://www.std.pmda.go.jp/stdDB/Data/MDStd/CerStd/Notif/K1100009_01_2016_en.pdf.

[54] Pharmaceuticals and Medical Devices Agency (PMDA) (2018). List of Certification Standards.

[55] Department of Therapeutic Goods Administration (TGA) Australian Regulatory Guidelines for Medical Devices (ARGMD). https://www.tga.gov.au/publication/australian-regulatory-guidelines-medical-devices-argmd.

[56] Standards Australia ISO 15197:2013. https://www.standards.org.au/standards-catalogue/international/iso-slash-tc--212/iso--15197-colon-2013.

[57] Department of Therapeutic Goods Administration (TGA) Medical Devices Regulation: An Introduction. http://www.tga.gov.au/sme-assist/medical-devices-regulation-introduction.

[58] MIT Carbon Nanotube Sensor Detects Glucose in Saliva. https://www.technologyreview.com/s/514456/carbon-nanotube-sensor-detects-glucose-in-saliva/.

[59] Jia J., Guan W., Sim M., Li Y., Li H. (2008). Carbon Nanotubes Based Glucose Needle-type Biosensor. Sensors.

[60] Zhou M., Wang Z., Wang X., Peng H., Li Q., Chen T. (2017). Chapter 5–Carbon Nanotubes for Sensing Applications. Industrial Applications of Carbon Nanotubes.

[61] Aslan K., Lakowicz J.R., Geddes C.D., Geddes C.D., Lakowicz J.R. (2006). Plasmonic Glucose Sensing. Glucose Sensing.

[62] Zhang W., Wang M.L. (2013). Saliva Glucose Monitoring System. U.S. patent.

[63] Li D.C., Wu J.W., Wu P., Lin Y., Sun Y.J., Zhu R., Yang J., Xu K.X. Glucose measurement using surface plasmon resonance sensor with affinity based surface modification by borate polymer. Proceedings of the 2015 Transducers-2015 18th International Conference on Solid-State Sensors, Actuators and Microsystems (TRANSDUCERS).

[64] Srivastava S.K., Verma R., Gupta B.D. Surface plasmon resonance based fiber optic glucose biosensor. Proceedings of the Third Asia Pacific Optical Sensors Conference.

[65] Li D., Su J., Yang J., Yu S., Zhang J., Xu K., Yu H. (2017). Optical surface plasmon resonance sensor modified by mutant glucose/galactose-binding protein for affinity detection of glucose molecules. Biomed. Opt. Express.

[66] Zeng S., Baillargeat D., Ho H.-P., Yong K.-T. (2014). Nanomaterials enhanced surface plasmon resonance for biological and chemical sensing applications. Chem. Soc. Rev..

[67] McShane M., Stein E., Cunningham D.D., Stenken J.A. (2009). Fluorescence-Based Glucose Sensors. In Vivo Glucose Sensing.

[68] Barone P.W., Parker R.S., Strano M.S. (2005). In Vivo Fluorescence Detection of Glucose Using a Single-Walled Carbon Nanotube Optical Sensor:  Design, Fluorophore Properties, Advantages, and Disadvantages. Anal. Chem..

[69] Klonoff D.C. (2012). Overview of Fluorescence Glucose Sensing: A Technology with a Bright Future. J. Diabetes Sci. Technol..

[70] Barone P.W., Strano M.S. (2009). Single Walled Carbon Nanotubes as Reporters for the Optical Detection of Glucose. J. Diabetes Sci. Technol..

[71] Chen L., Hwang E., Zhang J. (2018). Fluorescent Nanobiosensors for Sensing Glucose. Sensors.

[72] Szmacinski H., Lakowicz J.R. (1995). Fluorescence lifetime-based sensing and imaging. Sens. Actuators B Chem..

[73] Bambot S.B., Rao G., Romauld M., Carter G.M., Sipior J., Terpetchnig E., Lakowicz J.R. (1995). Sensing oxygen through skin using a red diode laser and fluorescence lifetimes. Biosens. Bioelectron..

[74] DiCesare N., Lakowicz J.R. (2001). Evaluation of two synthetic glucose probes for fluorescence-lifetime-based sensing. Anal. Biochem..

[75] Malik B.H., Coté G.L. (2010). Real-time, closed-loop dual-wavelength optical polarimetry for glucose monitoring. J. Biomed. Opt..

[76] Rawer R., Stork W., Kreiner C.F. (2004). Non-invasive polarimetric measurement of glucose concentration in the anterior chamber of the eye. Graefe’s Arch. Clin. Exp. Ophthalmol..

[77] Fercher A.F., Drexler W., Hitzenberger C.K., Lasser T. (2003). Optical coherence tomography - principles and applications. Rep. Prog. Phys..

[78] Lan Y.T., Kuang Y.P., Zhou L.P., Wu G.Y., Gu P.C., Wei H.J., Chen K. (2017). Noninvasive monitoring of blood glucose concentration in diabetic patients with optical coherence tomography. Laser Phys. Lett..

[79] Agelet L.E., Hurburgh C.R. (2010). A Tutorial on Near Infrared Spectroscopy and Its Calibration. Crit. Rev. Anal. Chem..

[80] Schaare P.N., Fraser D.G. (2000). Comparison of reflectance, interactance and transmission modes of visible-near infrared spectroscopy for measuring internal properties of kiwifruit (Actinidia chinensis). Postharvest Biol. Tech..

[81] Nicolaï B.M., Beullens K., Bobelyn E., Peirs A., Saeys W., Theron K.I., Lammertyn J. (2007). Nondestructive measurement of fruit and vegetable quality by means of NIR spectroscopy: A review. Postharvest Biol. Tech..

[82] Oliver N.S., Toumazou C., Cass A.E.G., Johnston D.G. (2009). Glucose sensors: a review of current and emerging technology. Diabet. Med..

[83] Maruo K., Oota T., Tsurugi M., Nakagawa T., Arimoto H., Tamura M., Ozaki Y., Yamada Y. (2006). New Methodology to Obtain a Calibration Model for Noninvasive Near-Infrared Blood Glucose Monitoring. Appl. Spectr..

[84] Khalil O.S. (2004). Non-Invasive Glucose Measurement Technologies: An Update from 1999 to the Dawn of the New Millennium. Diabetes Technol. Ther..

[85] Coates J. (1998). Vibrational Spectroscopy: Instrumentation for Infrared and Raman Spectroscopy. Appl. Spectr. Rev..

[86] Tura A., Maran A., Pacini G. (2007). Non-invasive glucose monitoring: Assessment of technologies and devices according to quantitative criteria. Diabetes Res. Clin. Pract..

[87] Liakat S., Bors K.A., Huang T.-Y., Michel A.P.M., Zanghi E., Gmachl C.F. (2013). In vitro measurements of physiological glucose concentrations in biological fluids using mid-infrared light. Biomed. Opt. Exp..

[88] MacKenzie H.A., Ashton H.S., Spiers S., Shen Y., Freeborn S.S., Hannigan J., Lindberg J., Rae P. (1999). Advances in Photoacoustic Noninvasive Glucose Testing. Clin. Chem..

[89] Liakat S., Bors K.A., Xu L., Woods C.M., Doyle J., Gmachl C.F. (2014). Noninvasive in vivo glucose sensing on human subjects using mid-infrared light. Biomed. Opt. Exp..

[90] von Lilienfeld-Toal H., Weidenmüller M., Xhelaj A., Mäntele W. (2005). A novel approach to non-invasive glucose measurement by mid-infrared spectroscopy: The combination of quantum cascade lasers (QCL) and photoacoustic detection. Vib. Spectr..

[91] Bumbrah G.S., Sharma R.M. (2016). Raman spectroscopy–Basic principle, instrumentation and selected applications for the characterization of drugs of abuse. Eg. J. Forensic Sci..

[92] Wiercigroch E., Szafraniec E., Czamara K., Pacia M.Z., Majzner K., Kochan K., Kaczor A., Baranska M., Malek K. (2017). Raman and infrared spectroscopy of carbohydrates: A review. Spectrochim. Acta Part A Mol. Biomol. Spectr..

[93] Xu Y., Ford J.F., Mann C.K., Vickers T.J., Brackett J.M., Cousineau K.L., Robey W.G. (1997). Raman measurement of glucose in bioreactor materials. Proc. SPIE.

[94] Pandey R., Paidi S.K., Valdez T.A., Zhang C., Spegazzini N., Dasari R.R., Barman I. (2017). Noninvasive Monitoring of Blood Glucose with Raman Spectroscopy. Acc. Chem. Res..

[95] Chaiken J., Deng B., Bussjager R.J., Shaheen G., Rice D., Stehlik D., Fayos J. (2010). Instrument for near infrared emission spectroscopic probing of human fingertips in vivo. Rev. Sci. Instrum..

[96] Koplik R. Infrared spectroscopy. https://web.vscht.cz/~poustkaj/EN%20ASFA%20AU%20Koplik_Infrared_spectroscopy.pdf.

[97] Alarousu E., Hast J.T., Kinnunen M.T., Kirillin M.Y., Myllyla R.A., Plucinski J., Popov A.P., Priezzhev A.V., Prykari T., Saarela J. Noninvasive glucose sensing in scattering media using OCT, PAS, and TOF techniques. Proceedings of the Saratov Fall Meeting 2003: Optical Technologies Biophysics and Medicine V.

[98] Withayachumnankul W., Naftaly M. (2014). Fundamentals of Measurement in Terahertz Time-Domain Spectroscopy. J. IR Millim. THz Waves.

[99] Cherkasova O.P., Nazarov M.M., Shkurinov A.P., Fedorov V.I. (2009). Terahertz spectroscopy of biological molecules. Radiophys. Quantum Electron..

[100] Cherkasova O., Nazarov M., Shkurinov A. (2016). Noninvasive blood glucose monitoring in the terahertz frequency range. Opt. Quantum Electron..

[101] Gusev S.I., Guseva V.A., Simonova A.A., Demchenko P.S., Sedykh E.A., Cherkasova O.P., Khodzitsky M.K. Application of terahertz pulsed spectroscopy for the development of non-invasive glucose measuring method. Proceedings of the 2017 Progress In Electromagnetics Research Symposium-Spring (PIERS).

[102] Torii T., Chiba H., Tanabe T., Oyama Y. (2017). Measurements of glucose concentration in aqueous solutions using reflected THz radiation for applications to a novel sub-THz radiation non-invasive blood sugar measurement method. Digit. Health.

[103] Malchoff C.D., Shoukri K., Landau J.I., Buchert J.M. (2002). A novel noninvasive blood glucose monitor. Diabetes Care.

[104] Klonoff D.C. (1997). Noninvasive Blood Glucose Monitoring. Diabetes Care.

[105] Buchert J.M. (2004). Thermal emission spectroscopy as a tool for noninvasive blood glucose measurements. Proc. SPIE.

[106] Cho O.K., Kim Y.O., Mitsumaki H., Kuwa K. (2004). Noninvasive Measurement of Glucose by Metabolic Heat Conformation Method. Clin. Chem..

[107] Tang F., Wang X., Wang D., Li J. (2008). Non-Invasive Glucose Measurement by Use of Metabolic Heat Conformation Method. Sensors.

[108] Sandeep K.V., Luong J.H.T. (2016). Point-of-Care Glucose Detection for Diabetic Monitoring and Management.

[109] Patel P., Hardik M., Patel P. (2013). A Review on Photoacoustic Spectroscopy. Int. J. Pharm. Erud..

[110] Tanaka Y., Tajima T., Seyama M. Differential photoacoustic spectroscopy with continuous wave lasers for non-invasive blood glucose monitoring. Proceedings of the Photons Plus Ultrasound: Imaging and Sensing 2018.

[111] Kottmann J., Rey J.M., Sigrist M.W. (2016). Mid-Infrared Photoacoustic Detection of Glucose in Human Skin: Towards Non-Invasive Diagnostics. Sensors.

[112] Sim J.Y., Ahn C.-G., Jeong E.-J., Kim B.K. (2018). In vivo Microscopic Photoacoustic Spectroscopy for Non-Invasive Glucose Monitoring Invulnerable to Skin Secretion Products. Sci. Rep..

[113] Pleitez M.A., Lieblein T., Bauer A., Hertzberg O., von Lilienfeld-Toal H., Mäntele W. (2013). Windowless ultrasound photoacoustic cell for in vivo mid-IR spectroscopy of human epidermis: Low interference by changes of air pressure, temperature, and humidity caused by skin contact opens the possibility for a non-invasive monitoring of glucose in the interstitial fluid. Rev. Sci. Instrum..

[114] Nakamura M., Tajima T., Ajito K., Koizumi H. Selectivity-enhanced glucose measurement in multicomponent aqueous solution by broadband dielectric spectroscopy. Proceedings of the 2016 IEEE MTT-S International Microwave Symposium (IMS).

[115] Bahar A.A.M., Zakaria Z., Isa A.A.M., Alahnomi R.A., Rahman N.A. Complex Permittivity Measurement Based on Planar Microfluidic Resonator Sensor. Proceedings of the 2018 18th International Symposium on Antenna Technology and Applied Electromagnetics (ANTEM).

[116] Shaker G., Smith K., Omer A.E., Liu S., Csech C., Wadhwa U., Safavi-Naeini S., Hughson R. (2018). Non-invasive monitoring of glucose level changes utilizing a mm-wave radar system. Int. J. Mob. Hum. Comput. Interact..

[117] Siegel P.H., Tang A., Virbila G., Kim Y., Chang M.C.F., Pikov V. Compact non-invasive millimeter-wave glucose sensor. Proceedings of the 2015 40th International Conference on Infrared, Millimeter, and Terahertz Waves (IRMMW-THz).

[118] Saha S., Cano-Garcia H., Sotiriou I., Lipscombe O., Gouzouasis I., Koutsoupidou M., Palikaras G., Mackenzie R., Reeve T., Kosmas P. (2017). A Glucose Sensing System Based on Transmission Measurements at Millimetre Waves using Micro strip Patch Antennas. Sci. Rep..

[119] Hofmann M., Fersch T., Weigel R., Fischer G., Kissinger D. A novel approach to non-invasive blood glucose measurement based on RF transmission. Proceedings of the 2011 IEEE International Symposium on Medical Measurements and Applications.

[120] Choi H., Naylon J., Luzio S., Beutler J., Birchall J., Martin C., Porch A. (2015). Design and In Vitro Interference Test of Microwave Noninvasive Blood Glucose Monitoring Sensor. IEEE Trans. Microw. Theory Tech..

[121] Zhang R., Qu Z., Jin H., Liu S., Luo Y., Zheng Y. Noninvasive Glucose Measurement by Microwave Biosensor with Accuracy Enhancement. Proceedings of the 2018 IEEE International Symposium on Circuits and Systems (ISCAS).

[122] Kim J., Babajanyan A., Hovsepyan A., Lee K., Friedman B. (2008). Microwave dielectric resonator biosensor for aqueous glucose solution. Rev. Sci. Instrum..

[123] Yilmaz T., Brizzi A., Foster R., Munoz M., Hao Y. A patch resonator for sensing blood glucose changes. Proceedings of the 2014 XXXIth URSI General Assembly and Scientific Symposium (URSI GASS).

[124] Gourzi M., Rouane A., Guelaz R., Alavi M.S., McHugh M.B., Nadi M., Roth P. (2005). Non-invasive glycaemia blood measurements by electromagnetic sensor: Study in static and dynamic blood circulation. J. Med. Eng. Tech..

[125] Melikyan H., Danielyan E., Kim S., Kim J., Babajanyan A., Lee J., Friedman B., Lee K. (2012). Non-invasive in vitro sensing of d-glucose in pig blood. Med. Eng. Phys..

[126] Weinzimer S.A. (2004). Analysis: PENDRA: The Once and Future Noninvasive Continuous Glucose Monitoring Device?. Diabetes Technol. Ther..

[127] Harman-Boehm I., Gal A., Raykhman A.M., Zahn J.D., Naidis E., Mayzel Y. (2009). Noninvasive Glucose Monitoring: A Novel Approach. J. Diabetes Sci. Technol..

[128] Kost J. (2002). Ultrasound-Assisted Insulin Delivery and Noninvasive Glucose Sensing. Diabetes Technol. Ther..

[129] Dubinsky T.J., Cuevas C., Dighe M.K., Kolokythas O., Hwang J.H. (2008). High-Intensity Focused Ultrasound: Current Potential and Oncologic Applications. Am. J. Roentgenol..

[130] Garg S.K., Potts R.O., Ackerman N.R., Fermi S.J., Tamada J.A., Chase H.P. (1999). Correlation of fingerstick blood glucose measurements with GlucoWatch biographer glucose results in young subjects with type 1 diabetes. Diabetes Care.

[131] Potts R.O., Tamada J.A., Tierney M.J. (2002). Glucose monitoring by reverse iontophoresis. Diabetes/Metab. Res. Rev..

[132] Cooke D., Hurel S.J., Casbard A., Steed L., Walker S., Meredith S., Nunn A.J., Manca A., Sculpher M., Barnard M. (2009). Randomized controlled trial to assess the impact of continuous glucose monitoring on HbA1c in insulin-treated diabetes (MITRE Study). Diabetic Med..

[133] Gandrud L.M., Paguntalan H.U., Van Wyhe M.M., Kunselman B.L., Leptien A.D., Wilson D.M., Eastman R.C., Buckingham B.A. (2004). Use of the Cygnus GlucoWatch biographer at a diabetes camp. Pediatrics.

[134] Wentholt I.M.E., Hoekstra J.B.L., Zwart A., DeVries J.H. (2005). Pendra goes Dutch: lessons for the CE mark in Europe. Diabetologia.

[135] MIT Technology Review Blood Sugar Crash. https://www.technologyreview.com/s/529026/blood-sugar-crash/.

[136] Segman Y. (2018). Device and Method for Noninvasive Glucose Assessment. J. Diabetes Sci. Technol..

[137] Pfützner A., Strobl S., Demircik F., Redert L., Pfützner J., Pfützner A.H., Lier A. (2018). Evaluation of a New Noninvasive Glucose Monitoring Device by Means of Standardized Meal Experiments. J. Diabetes Sci. Technol..

[138] Pfützner A. (2017). Evaluation of the CNOGA COMBO GLUCOMETER and the MTX Non-Invasive Body Signaling Device During a Standardized Meal Test in Patients with Diabetes Mellitus and in Healthy Subjects.

[139] Segman Y. (2005). Optical sensor device and image processing unit for measuring chemical concentrations, chemical saturations and biophysical parameters. U.S. Patent.

[140] Amir O., Weinstein D., Zilberman S., Less M., Perl-Treves D., Primack H., Weinstein A., Gabis E., Fikhte B., Karasik A. (2007). Continuous Noninvasive Glucose Monitoring Technology Based on “Occlusion Spectroscopy”. J. Diabetes Sci. Technol..

[141] Fine I., Ma’Ayan L. (2003). Glucose level control method and system. U.S. Patent.

[142] Eisen L., Fine I., Goldinov L. (2011). Wearable pulse oximetry device. U.S. Patent.

[143] Offord C. Will the Noninvasive Glucose Monitoring Revolution Ever Arrive?. https://www.the-scientist.com/news-analysis/will-the-noninvasive-glucose-monitoring-revolution-ever-arrive-30754.

[144] World Global Network. Science behind the HELO. https://www.wearablelifestyles.net/science-behind-the-helo/.

[145] Pfützner A., Sachsenheimer D., Mills L., Deakin S., Moore K., Saini S., MacRury S. (2012). Evaluation of the Non-Invasive Glucose Monitoring Device GlucoTrack in Patients with Type 2 Diabetes and Subjects with Prediabetes.

[146] Harman-Boehm I., Gal A., Raykhman A.M., Naidis E., Mayzel Y. (2010). Noninvasive Glucose Monitoring: Increasing Accuracy by Combination of Multi-Technology and Multi-Sensors. J. Diabetes Sci. Technol..

[147] MediWise GlucoWise. http://www.gluco-wise.com/.

[148] Nemaura Medical Nemaura Announces Positive Results for Its SugarBEAT^®^ European Clinical Program. http://nemauramedical.com/nemaura-announces-positive-results-sugarbeat-european-clinical-program/.

[149] Saur N.M., England M.R., Menzie W., Melanson A.M., Trieu M.-Q., Berlin J., Hurley J., Krystyniak K., Kongable G.L., Nasraway S.A. (2014). Accuracy of a Novel Noninvasive Transdermal Continuous Glucose Monitor in Critically Ill Patients. J. Diabetes Sci. Technol..

[150] Hadar E., Chen R., Toledano Y., Tenenbaum-Gavish K., Atzmon Y., Hod M. (2018). Noninvasive, continuous, real-time glucose measurements compared to reference laboratory venous plasma glucose values. J. Matern. Fetal Neonatal Med..

[151] Optronics Online QST Developed a Non-Invasive Blood Glucose Measurement Technique with a Mid-Infrared Laser. http://www.optronics-media.com/news/20170822/47807/.

[152] PKvitality K’Watch Continuous Glucose Monitoring (CGM) Device. https://www.pkvitality.com/wp-content/uploads/2018/11/PKVITALITY-KWatch-CGM-21112018-EN.pdf.

[153] PKvitality K’Watch Glucose CGM Reinvented. https://www.pkvitality.com/ktrack-glucose/.

[154] DeHennis A., Tankiewicz S., Whitehurst T. (2015). Analyte sensor. U.S. Patent.

[155] Jafri R.Z., Balliro C.A., El-Khatib F., Maheno M., Hillard M.A., Donovan A.J., Selagamsetty R., Zheng H.U.I., Damiano E., Russell S.J. (2018). A Three-Way Accuracy Comparison of the Dexcom G5, Abbott Freestyle Libre Pro, and Senseonics Eversense CGM Devices in an Outpatient Study of Subjects with Type 1 Diabetes. Diabetes.

[156] Senseonics Eversense User Guide. https://www.eversensediabetes.com/wp-content/uploads/2018/08/LBL-1602-01-001-Rev-D_Eversense-User-Guide_mgdL_R1-2.pdf.

[157] Tang F., You Z., Wang X., Li Y., Yan Y., Fan Z. (2011). Non-Invasive Blood Glucose Detector Based on Metabolic Heat-Optical Method. Chinese Patent.

[158] Siegel P.H., Lee Y., Pikov V. Millimeter-wave non-invasive monitoring of glucose in anesthetized rats. Proceedings of the 2014 39th International Conference on Infrared, Millimeter, and Terahertz waves (IRMMW-THz).

[159] Siegel P.H., Tang A., Kim R., Virbila G., Chang F., Pikov V. Noninvasive in vivo millimeter-wave measurements of glucose: First results in human subjects. Proceedings of the 2017 42nd International Conference on Infrared, Millimeter, and Terahertz Waves (IRMMW-THz).

[160] Chen L., Tse W.H., Chen Y., McDonald M.W., Melling J., Zhang J. (2017). Nanostructured biosensor for detecting glucose in tear by applying fluorescence resonance energy transfer quenching mechanism. Biosens. Bioelectr..

[161] Monte-Moreno E. (2011). Non-invasive estimate of blood glucose and blood pressure from a photoplethysmograph by means of machine learning techniques. Artif. Intell. Med..

[162] Philip L.A., Rajasekaran K., Jothi E.S.J. Continous monitoring of blood glucose using photophlythesmograph signal. Proceedings of the 2017 International Conference on Innovations in Electrical, Electronics, Instrumentation and Media Technology (ICEEIMT).

[163] Kino S., Omori S., Katagiri T., Matsuura Y. (2016). Hollow optical-fiber based infrared spectroscopy for measurement of blood glucose level by using multi-reflection prism. Biomed. Opt. Exp..

[164] Lundsgaard-Nielsen S.M., Pors A., Banke S.O., Henriksen J.E., Hepp D.K., Weber A. (2018). Critical-depth Raman spectroscopy enables home-use non-invasive glucose monitoring. PLoS ONE.

[165] Chen T.-L., Lo Y.-L., Liao C.-C., Phan Q.-H. (2018). Noninvasive measurement of glucose concentration on human fingertip by optical coherence tomography. J. Biomed. Opt..

[166] Hofmann M., Fischer G., Weigel R., Kissinger D. (2013). Microwave-Based Noninvasive Concentration Measurements for Biomedical Applications. IEEE Trans. Microw. Theory Tech..

[167] Choi H., Luzio S., Beutler J., Porch A. Microwave noninvasive blood glucose monitoring sensor: Human clinical trial results. Proceedings of the 2017 IEEE MTT-S International Microwave Symposium (IMS).

[168] Lipani L., Dupont B.G.R., Doungmene F., Marken F., Tyrrell R.M., Guy R.H., Ilie A. (2018). Non-invasive, transdermal, path-selective and specific glucose monitoring via a graphene-based platform. Nat. Nanotechnol..

[169] Vega K., Jiang N., Liu X., Kan V., Barry N., Maes P., Yetisen A., Paradiso J. The dermal abyss: interfacing with the skin by tattooing biosensors. Proceedings of the 2017 ACM International Symposium on Wearable Computers (ISWC’17).

[170] Park J., Kim J., Kim S.-Y., Cheong W.H., Jang J., Park Y.-G., Na K., Kim Y.-T., Heo J.H., Lee C.Y. (2018). Soft, smart contact lenses with integrations of wireless circuits, glucose sensors, and displays. Sci. Adv..

[171] Badugu R., Reece E.A., Lakowicz J.R. (2018). Glucose-sensitive silicone hydrogel contact lens toward tear glucose monitoring. J. Biomed. Opt..

[172] Ribet F., Stemme G., Roxhed N. (2018). Real-time intradermal continuous glucose monitoring using a minimally invasive microneedle-based system. Biomed. Microdevices.

[173] Gamsey S., Suri J.T., Wessling R.A., Singaram B. (2006). Continuous Glucose Detection Using Boronic Acid-Substituted Viologens in Fluorescent Hydrogels:  Linker Effects and Extension to Fiber Optics. Langmuir.

[174] Gamsey S., Bernat V., Kutyavin A., Clary J.W., Pradhan S. (2016). Near-IR glucose sensors. U.S. Patent.

[175] Larin K.V., Eledrisi M.S., Motamedi M., Esenaliev R.O. (2002). Noninvasive Blood Glucose Monitoring With Optical Coherence Tomography. Diabetes Care.

[176] Palerm C.C., Bequette B.W. (2007). Hypoglycemia Detection and Prediction Using Continuous Glucose Monitoring—A Study on Hypoglycemic Clamp Data. J. Diabetes Sci. Techol..

[177] Mahmoudi Z., Dencker Johansen M., Christiansen J.S., Hejlesen O.K. (2013). A Multistep Algorithm for Processing and Calibration of Microdialysis Continuous Glucose Monitoring Data. Diabetes Technol. Ther..

[178] Facchinetti A. (2016). Continuous Glucose Monitoring Sensors: Past, Present and Future Algorithmic Challenges. Sensors.

[179] Barceló-Rico F., Bondia J., Díez J.L., Rossetti P. (2011). A Multiple Local Models Approach to Accuracy Improvement in Continuous Glucose Monitoring. Diabetes Technol. Ther..

[180] Kirchsteiger H., Zaccarian L., Renard E., Re L.D. (2015). LMI-Based Approaches for the Calibration of Continuous Glucose Measurement Sensors. IEEE J. Biomed. Health Inf..

[181] Sparacino G., Zanderigo F., Maran A., Cobelli C. (2006). Continuous glucose monitoring and hypo/hyperglycaemia prediction. Diabetes Res. Clin. Pract..

[182] Reifman J., Rajaraman S., Gribok A., Ward W.K. (2007). Predictive Monitoring for Improved Management of Glucose Levels. J. Diabetes Sci. Technol..

[183] Pérez-Gandía C., Facchinetti A., Sparacino G., Cobelli C., Gómez E.J., Rigla M., de Leiva A., Hernando M.E. (2010). Artificial Neural Network Algorithm for Online Glucose Prediction from Continuous Glucose Monitoring. Diabetes Technol. Ther..

[184] Zanon M., Sparacino G., Facchinetti A., Talary S.M., Mueller M., Caduff A., Cobelli C. (2013). Non-Invasive Continuous Glucose Monitoring with Multi-Sensor Systems: A Monte Carlo-Based Methodology for Assessing Calibration Robustness. Sensors.

[185] Eadie M., Steele R. Non-invasive Blood Glucose Monitoring and Data Analytics. Proceedings of the ICCDA’17.

[186] Sandham W., Nikoletou D., Hamilton D., Paterson K., Japp A., Macgregor C. Blood glucose prediction for diabetes therapy using a recurrent artificial neural network. Proceedings of the EUSIPCO.

[187] Tresp V., Briegel T., Moody J. (1999). Neural-network models for the blood glucose metabolism of a diabetic. IEEE Trans. Neural Netw..

[188] Mougiakakou S.G., Prountzou K., Nikita K.S. A Real Time Simulation Model of Glucose-Insulin Metabolism for Type 1 Diabetes Patients. Proceedings of the 2005 IEEE Engineering in Medicine and Biology 27th Annual Conference.

[189] Smith J. The Pursuit of Noninvasive Glucose: Hunting the Deceitful Turkey.

[190] Purvinis G., Cameron B.D., Altrogge D.M. (2011). Noninvasive polarimetric-based glucose monitoring: an in vivo study. J. Diabetes Sci. Techol..

[191] Winkler A.M., Bonnema G.T., Barton J.K. (2011). Optical polarimetry for noninvasive glucose sensing enabled by Sagnac interferometry. Appl. Opt..

[192] Yao H., Shum A.J., Cowan M., Lähdesmäki I., Parviz B.A. (2011). A contact lens with embedded sensor for monitoring tear glucose level. Biosens. Bioelectron..

[193] Chu M.X., Miyajima K., Takahashi D., Arakawa T., Sano K., Sawada S.-i., Kudo H., Iwasaki Y., Akiyoshi K., Mochizuki M. (2011). Soft contact lens biosensor for in situ monitoring of tear glucose as non-invasive blood sugar assessment. Talanta.

[194] Zhang J., Hodge W.G. (2008). Contact lens integrated with a biosensor for the detection of glucose and other components in tears. U.S. Patent.

[195] Ruan J.-L., Chen C., Shen J.-H., Zhao X.-L., Qian S.-H., Zhu Z.-G. (2017). A Gelated Colloidal Crystal Attached Lens for Noninvasive Continuous Monitoring of Tear Glucose. Polymers.

[196] Elsherif M., Hassan M.U., Yetisen A.K., Butt H. (2018). Wearable Contact Lens Biosensors for Continuous Glucose Monitoring Using Smartphones. ACS Nano.

[197] Tseng R.C., Chen C.-C., Hsu S.-M., Chuang H.-S. (2018). Contact-Lens Biosensors. Sensors.

